# Morbidity after surgical management of cervical cancer in low and middle income countries: A systematic review and meta-analysis

**DOI:** 10.1371/journal.pone.0217775

**Published:** 2019-07-03

**Authors:** Emma R. Allanson, Aime Powell, Max Bulsara, Hong Lim Lee, Lynette Denny, Yee Leung, Paul Cohen

**Affiliations:** 1 Division of Obstetrics and Gynaecology, Faculty of Medicine and Health Sciences, University of Western Australia, Crawley, WA, Australia; 2 Institute for Health Research, University of Notre Dame Australia, Fremantle, WA, Australia; 3 Obstetrics and Gynaecology, Joondalup Health Campus, Joondalup, WA, Australia; 4 Department Obstetrics and Gynaecology, University of Cape Town, Cape Town, South Africa; 5 South African Medical Research Council Gynaecological Cancer Research Centre, Cape Town, South Africa; 6 Department of Gynaecological Oncology, Bendat Family Comprehensive Cancer Centre, St John of God, Subiaco, WA, Australia; Tata Memorial Centre, INDIA

## Abstract

**Objective:**

To investigate morbidity for patients after the primary surgical management of cervical cancer in low and middle-income countries (LMIC).

**Methods:**

The Pubmed, Cochrane, the Cochrane Central Register of Controlled Trials, Embase, LILACS and CINAHL were searched for published studies from 1^st^ Jan 2000 to 30^th^ June 2017 reporting outcomes of surgical management of cervical cancer in LMIC. Random-effects meta-analytical models were used to calculate pooled estimates of surgical complications including blood transfusions, ureteric, bladder, bowel, vascular and nerve injury, fistulae and thromboembolic events. Secondary outcomes included five-year progression free (PFS) and overall survival (OS).

**Findings:**

Data were available for 46 studies, including 10,847 patients from 11 middle income countries. Pooled estimates were: blood transfusion 29% (95%CI 0.19–0.41, P = 0.00, I^2^ = 97.81), nerve injury 1% (95%CI 0.00–0.03, I^2^ 77.80, P = 0.00), bowel injury, 0.5% (95%CI 0.01–0.01, I^2^ = 0.00, P = 0.77), bladder injury 1% (95%CI 0.01–0.02, P = 0.10, I^2^ = 32.2), ureteric injury 1% (95%CI 0.01–0.01, I^2^ 0.00, P = 0.64), vascular injury 2% (95% CI 0.01–0.03, I^2^ 60.22, P = 0.00), fistula 2% (95%CI 0.01–0.03, I^2^ = 77.32, P = 0.00,), pulmonary embolism 0.4% (95%CI 0.00–0.01, I^2^ 26.69, P = 0.25), and infection 8% (95%CI 0.04–0.12, I^2^ 95.72, P = 0.00). 5-year PFS was 83% for laparotomy, 84% for laparoscopy and OS was 85% for laparotomy cases and 80% for laparoscopy.

**Conclusion:**

This is the first systematic review and meta-analysis of surgical morbidity in cervical cancer in LMIC, which highlights the limitations of the current data and provides a benchmark for future health services research and policy implementation.

## Introduction

Cervical cancer is the third most common malignancy in women worldwide and performs poorly in all objective measurements of outcomes in less developed countries[1]. The disease is a notable example of an extreme global health disparity with almost all cervical cancers, and the deaths caused by them, occurring in low and middle-income countries (LMIC)[2]. Contributors to this inequity are complex and multifaceted and include insufficient access to HPV vaccines and screening, and lack of trained health care professionals, radiation services and infrastructure, that prohibit reductions in cervical cancer incidence and mortality within these countries [3].

Opportunities to prevent and control cervical cancer arise from the human Papillomavirus (HPV) vaccine and adopting an organised approach to cervical screening. Whilst there have been global efforts towards this, for the half a million women with cervical cancer in LMIC, access to appropriate surgical, radiotherapy, and chemotherapy treatments are critical to reducing the burden of disease in LMIC and should remain a focus of global healthcare [4–7].

Surgical care should be a fundamental component of all health systems regardless of development level. This is particularly relevant in LMIC, where surgeons may be the sole physician involved in delivery of cancer care[3]. The delivery of high-quality surgical services that treat cervical cancer will prevent deaths, limit disability and suffering and promote economic growth (15*)*. Research investigating surgical outcomes is critical to improve patient health outcomes. Practitioner adherence to protocols should be tracked and adverse events openly reviewed (15). Currently patients in LMIC are over-represented in reports of global rates of adverse surgical outcomes[8], with women’s cancers having been long neglected in these settings[9]. Allowing for variations in reporting, overall complication rates for radical hysterectomy are, often from single centres[10], reported at up to 37% [11, 12], and rates of individual complications, e.g. voiding dysfunction, are reported at up to 42%[13, 14]. However, these reports largely come from high-income countries.

Data regarding cervical cancer surgical outcomes in LMIC are lacking and a baseline measure is necessary in order to advocate for ongoing quality care in these countries. Therefore, this systematic review and meta-analysis aims to report the morbidity of cervical cancer surgery in LMIC and provides a benchmark for future research initiatives.

## Materials and methods

This systematic review and meta-analysis was performed in accordance with the Preferred Reporting Items for Systematic reviews and Meta-Analysis (PRISMA) guidelines [15]. We searched Pubmed, Cochrane, the Cochrane Central Register of Controlled Trials, Embase, LILACS, and CINAHL to identify all relevant articles published January 1, 2000, to June 30, 2017, without language restriction. The year 2000 was chosen as the limit of the search for both pragmatic reasons and in order that the findings were reflective of current practice.

We applied a search strategy combining relevant terms, including: Uterine Cervical Neoplasms/surgery AND ((Hysterectomy/adverse effects*) OR (Laparoscopy/adverse effects) OR (Postoperative Complications*) OR (Operative Time) OR (Perioperative Period*) OR (Blood Loss, Surgical) OR (Intraoperative Complications) OR (Ureter) OR (Cystotomy) OR (Intestines/surgery) OR (Urinary Bladder, Neurogenic) OR (Lymphocele) OR (Fistula)) OR (Hystere* OR Laparo* OR Complication* OR Operati* OR Blood OR Morbidity OR Bowel injury OR Pelvic abscess OR Vascular injury OR Lymph*)). The full search strategy can be found in S1 Table. Reference lists from full text articles identified following the title and abstract screen were hand searched for any additional references relevant to the review question.

### Inclusion and exclusion criteria

Included participants were women in a LMIC [16], with a diagnosis of cervical cancer undergoing surgical intervention. The exception to this was for studies from Taiwan, for which the data (for most indicators) is added to high-income country aggregates [17]. Surgical management was defined as a simple or radical hysterectomy (+/- pelvic lymphadenectomy or pelvic lymph node dissection). All studies were required to have a minimum of 20 cervical cancer patients (at a minimum rate of 5 patients per year included, to avoid overestimation of complications, such as from small case series) and report on at least two surgical complications. Studies were excluded if there were no data available on the individual surgical complications.

**Fig 1:**
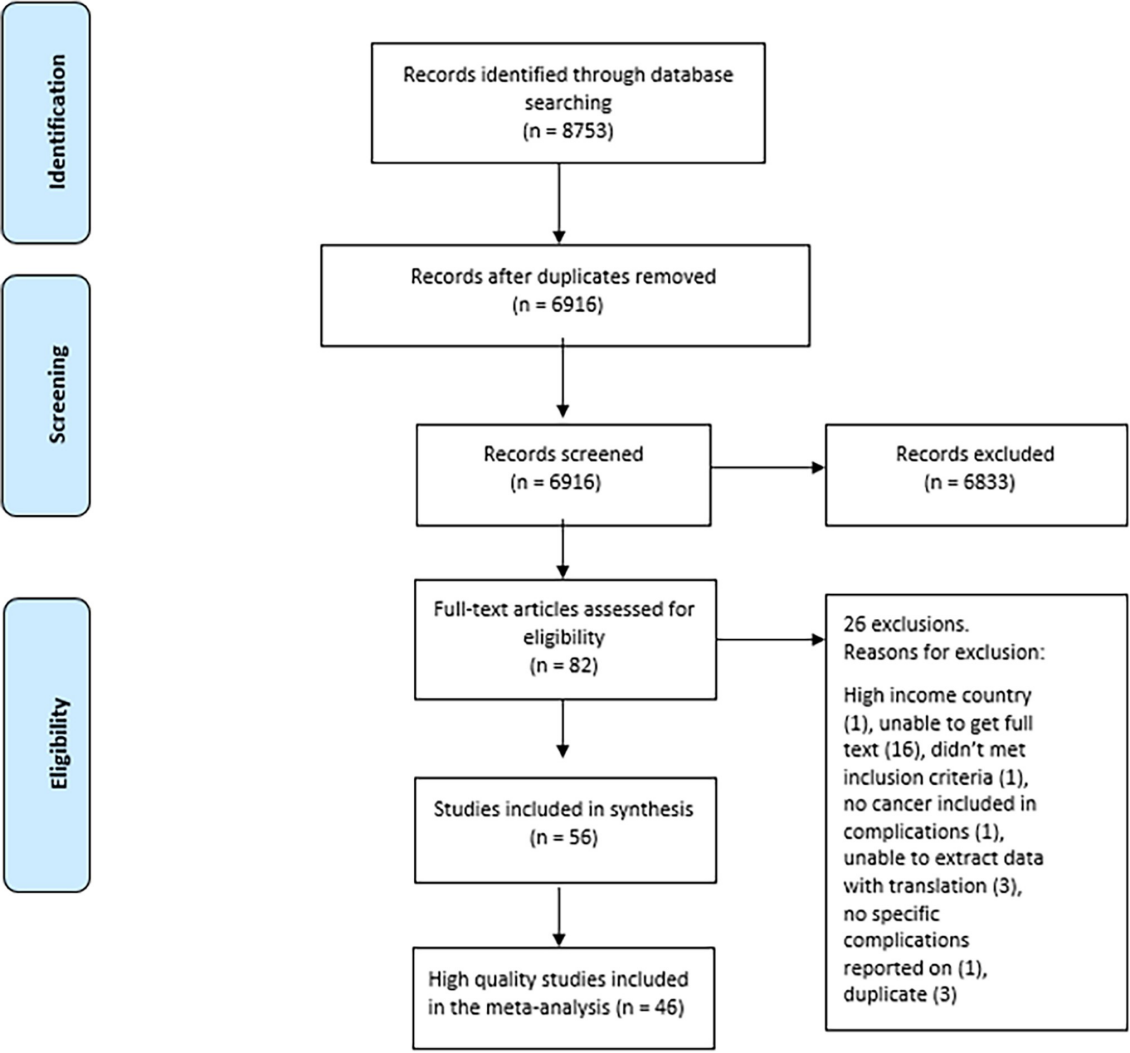
PRISMA flowchart.

Two investigators independently screened the titles and abstracts of articles retrieved from the literature search, and the full texts of potentially eligible articles were obtained and further assessed for final inclusion (Refer to Fig 1). Disagreements were resolved through consensus.

### Outcome measures

The primary measure was to evaluate the reported prevalence of surgical morbidity for cervical cancer patients treated with surgery in LMIC. Secondary outcome measures included progression free survival (PFS) (the time from surgery to the first relapse, progression of existing disease or the last contact date) and OS (the interval from surgery to death (from any cause).

### Classification of surgical outcomes

Complications were extracted as reported by the authors of each study (S1 File). Four authors (EA, AP, PC, YL) reviewed these in a collaborative session and agreed on the grouping of complications for analysis. Some complications were not considered surgical morbidity (for example, “IUD withdrawal difficulty”) and so were excluded from any further analysis. A pragmatic approach was adopted, and complications that were only reported once and/or unlikely to require acute surgical management (e.g. lymphocyst) were excluded from the meta-analysis.

We have presented results for blood transfusions, bladder injury, ureteric injury, bowel injury, nerve injury, vascular injury, conversion to laparotomy (for planned laparoscopic cases), fistula, pulmonary embolism, combined thromboembolic events (pulmonary embolism and venous thromboembolism), and infectious morbidity. Infectious morbidity included wound infection, post-operative infection, urinary tract infection, urosepsis, vaginal cuff infection, febrile morbidity, necrotising fasciitis, pelvic cellulitis, pneumonia, pyelonephritis, and pelvic abscess.

### Assessment of methodological and reporting quality

Methodological quality was assessed using the Cochrane Risk of Bias tool for randomized trials. For non-randomised studies, the Newcastle-Ottawa Scale (NOS) was adapted and used for the cohort studies [18]. We also included three elements (all outcome measures reported, methods of assessment for outcome provided, authors discuss potential sources of bias) from the Strengthening the Reporting of Observational Studies in Epidemiology (STROBE) quality assessment tool when assessing cohort studies [19–21].

### Meta-analysis

Studies were included in the meta-analysis if they were assessed as high quality. Randomised control trials were excluded from the analysis if they had a high risk of bias in any domain [22]. Cohort studies were excluded if they had a Newcastle-Ottawa score of less than 7 [23].

### Statistical analysis

Summary estimates and 95% confidence intervals (CIs) for various complication types were obtained with a random effects model by pooling proportions from each study[24]. A random effects model for pooling proportions was used to account for variability in the effect estimates and subgroup meta-analyses were used to distinguish the respective treatments performed (i.e. laparotomy vs. laparoscopic surgery)[25]. The percentage of total variation across studies due to heterogeneity was evaluated by the I2 measure[26, 27]. Forrest plots were drawn showing the variation of the specific surgical complication rate among all studies together with the pooled estimate measure[28, 29]. Given the large number of studies included from China, a sensitivity analysis was conducted that excluded all Chinese studies. Funnel plots were used to assess the potential role of publication bias. Egger’s test was used as a formal test of funnel plot asymmetry and publication bias[30]. Stata, version 15.0 (Stata Corp., College Station, TX, USA), was used for statistical analysis[31].

### Registration

The protocol for this review was registered PROSPERO registration number CRD42017057205.

## Results

**Fig 2:**
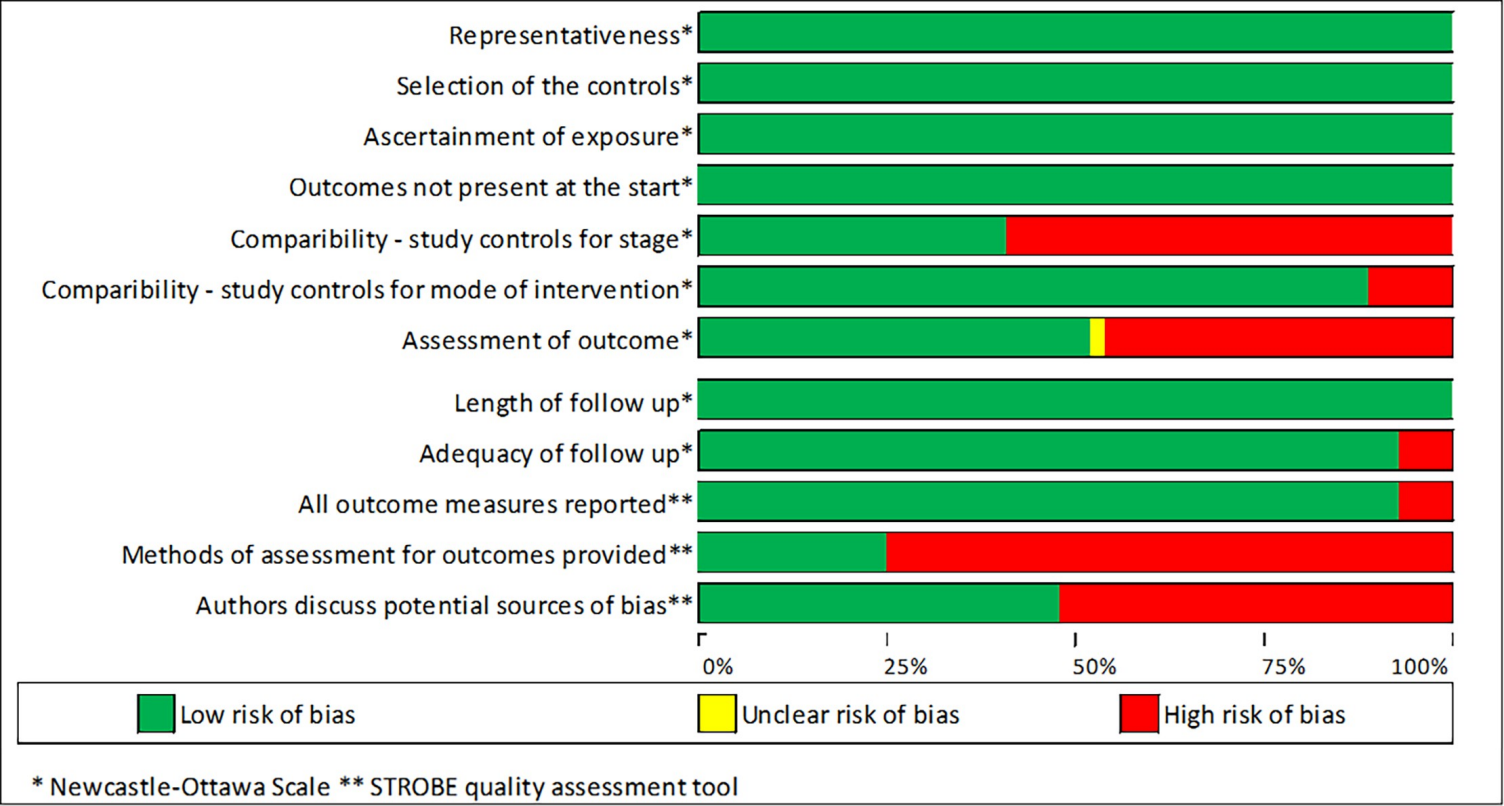
Risk of bias assessment in the included cohort studies.

**Fig 3:**
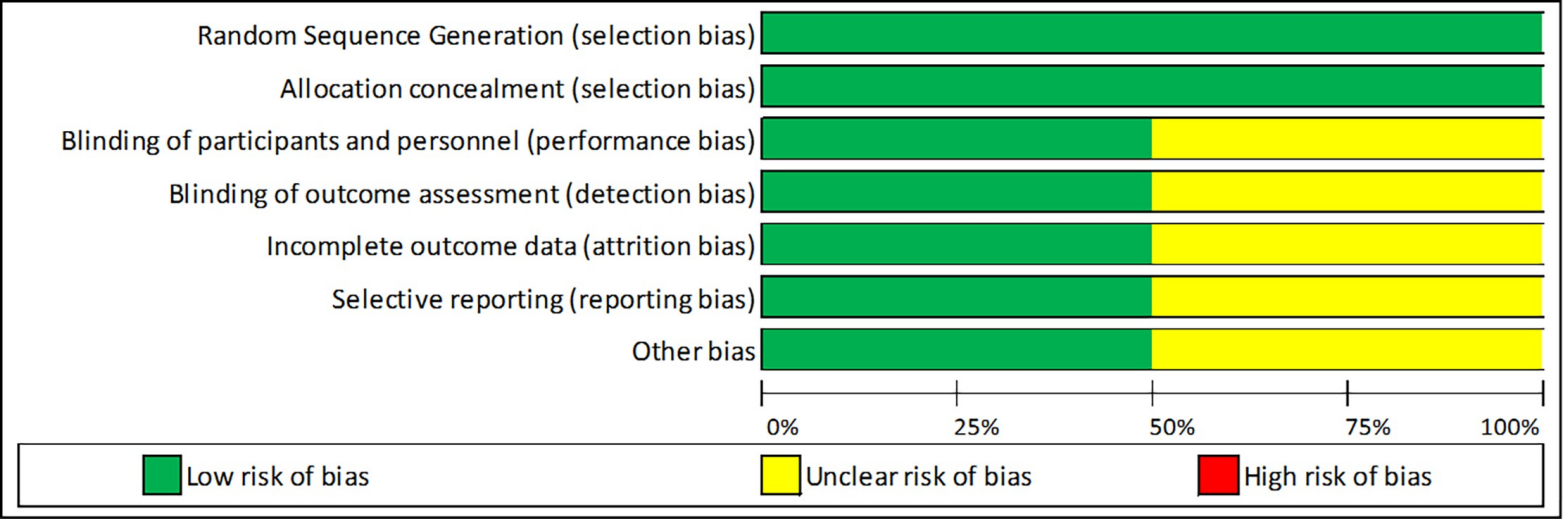
Risk of bias assessment in the included randomised studies.

Fig 1 shows the selection of studies. A total number of 56 articles met the study inclusion criteria. Two studies which reported on trachelectomy only were excluded from the meta-analysis[32, 33]. One high quality study included stage 4 cancers (the remainder included only early stage disease 1A-2B) and was excluded from the analysis. Figs 2 and 3 present the risk of bias assessment in the included high quality studies. Studies were high quality in most domains; however, it was common that stage of disease was not controlled for, and that the assessment of the outcome and the method of assessment were not adequately described.

**Fig 4:**
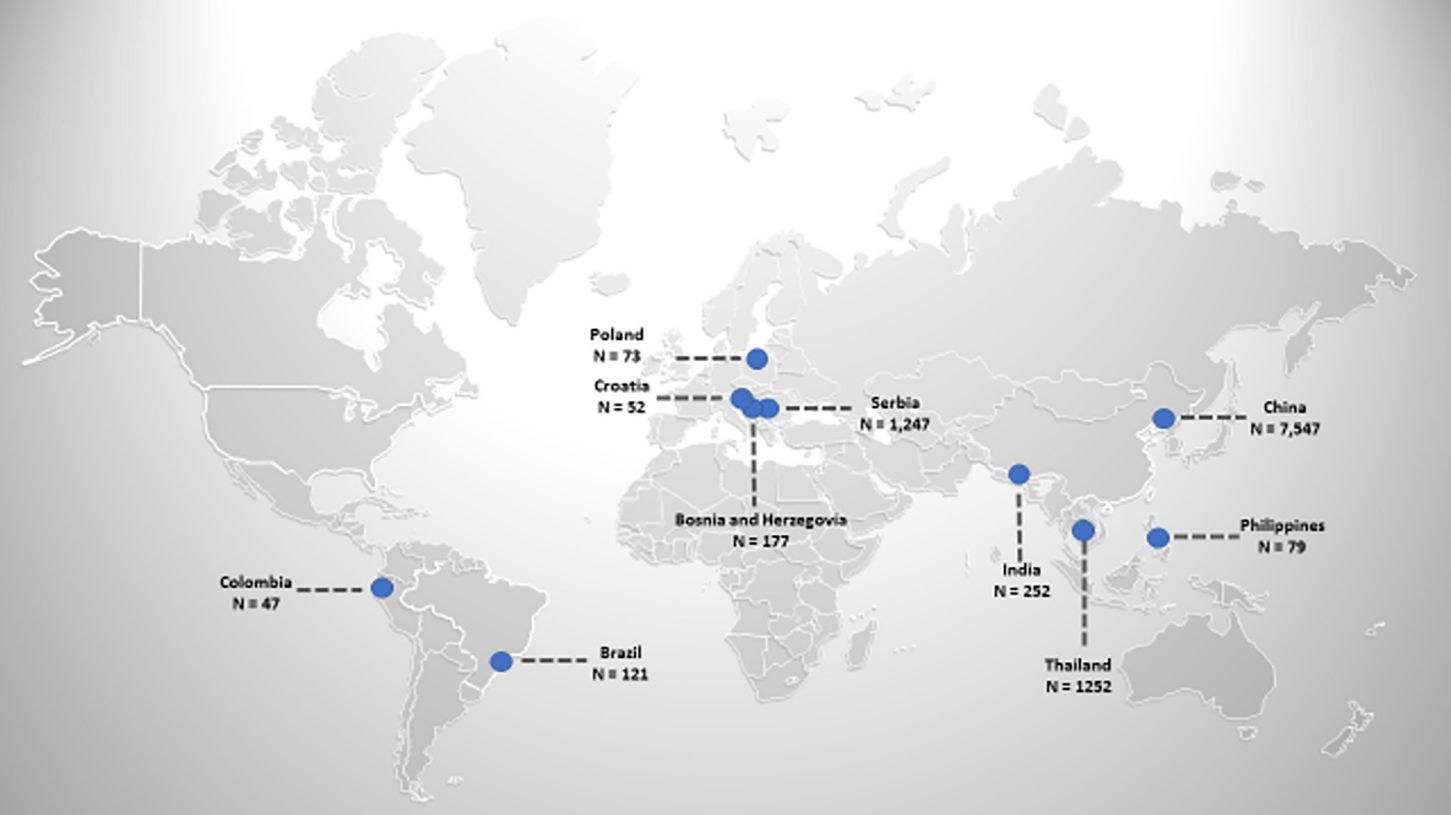
Distribution of included patients in LMIC.

After excluding low-quality studies, data were available for 46 studies [34–79], for 10,847 patients (from 2 randomized trials, 9 prospective and 35 retrospective studies) from 11 middle-income countries (Fig 4). Low income country studies from Asia, Africa, South America and Russia were excluded studies as they were assessed as low quality studies. Characteristics of included studies are shown in Table 1. The median age of participants was in the 5^th^ or 6^th^ decade in all but one study[40]. The study authors variably described surgical approaches, and while inclusion criteria specified the need for confirmed malignancy, this was rarely confirmed with post-operative histology. 10814 patients had a radical hysterectomy. 33 had a simple hysterectomy. 2,187 patients had a laparoscopic procedure, and 8,660 patients had a laparotomy. Surgery was performed in a wide variety of centres, from general hospitals to specialised cancer units. 23 high quality studies included 1663/4572 patients (36.4%) that had neoadjuvant treatment, both chemotherapy and / or radiotherapy. The neoadjuvant treatment regimens were varied and are not reported here.

**Table 1:** Characteristics of included high quality studies.

Reference	Country	Study design	Total study cohort	FIGO stage	Type of surgery*	Mode(s) of surgery
Srisomboon, 2002 (24)	Thailand	RCT	100	1A2-2A	RH and PLND	Laparotomy
Cai, 2006 (25)	China	RCT	106	1B	RH and PLND	Laparotomy
Cao, 2006 [36]	China	Retrospective cohort	139	1A-2B	RH and PLND	Laparotomy
Coruasic, 2007 [37]	Croatia	Cohort**	52	1A-1B	RH	Laparoscopy, laparotomy
Li, 2007 [38]	China	Retrospective cohort	125	1B-2A	RH and PLND	Laparoscopy, laparotomy
Likic-Ladevic, 2007 [39]	Serbia	Prospective cohort	536	1B-2B	RH	Laparotomy
Puntambekar, 2007 [40]	India	Retrospective cohort	252	1A2-1B1	RH and PLND	Laparoscopy
Xu, 2007 [41]	China	Retrospective cohort	317	1B-2A	RH and PLND +/- aortic LND	Laparoscopy
Chen, 2008 [42]	China	Retrospective cohort	295	1A2-2B	RH and PLND +/- aortic LND	Laparoscopy
Kietpeerakool, 2008 [43]	Thailand	Retrospective cohort	357	1B2-2B	RH and PLND	Laparotomy
Likic, 2008 [44]	Serbia	Prospective cohort	536	1B	RH	Laparotomy
Liu, 2008[96]	China	Retrospective cohort	143	1A-2B	RH and PLND	Laparotomy
Cai, 2009 [45]	China	Prospective cohort	480	1B-2A	RH	Laparotomy
Ju, 2009 [46]	China	Retrospective cohort	93	1A-2B	RH	Laparotomy
Manchana, 2009 [47]	Thailand	Retrospective cohort	281	1B-2A	RH	Laparotomy
Cai, 2010 [48]	China	Retrospective cohort	372	1B-2A	RH	Laparotomy
Espino-Strebel, 2010 [49]	Philippines	Retrospective cohort	79	1-2A	RH	Laparotomy
Zhu, 2010 [50]	China	Retrospective cohort	132	IB-2A	RH	Laparotomy
Bezerra, 2011 [51]	Brazil	Retrospective cohort	88	1A-2A	RH and PLND	Laparotomy
Hou, 2011 [52]	China	Prospective cohort	63	1A-2B	RH and PLND	Laparoscopy, laparotomy
Li, 2011 [53]	China	Prospective cohort	73	1B2-2A	RH	Laparotomy
Lucic, 2011 [54]	Bosnia and Herzegovina	Retrospective cohort	177	1B1-2B	RH	Laparotomy
Yan, 2011 [55]	China	Retrospective cohort	240	1A2-2B	RH and PLND	Laparoscopy
Zheng, 2011 [56]	China	Retrospective cohort	960	1B-2B	RH	Laparotomy
Zhou, 2011 [57]	China	Retrospective cohort	80	1B2-2A	RH and PLND	Laparotomy
Zhu, 2011 [58]	China	Cohort**	61	1B1-2A	RH	Laparotomy
Achavanuntakul, 2012 [59]	Thailand	Retrospective cohort	456	1A2-2A	RH and PLND	Laparotomy
Li, 2012 [60]	China	Retrospective cohort	391	1A2-2B	RH and PLND +/- aortic LND	Laparotomy
Pareja, 2012 [61]	Colombia	Retrospective cohort	47	1A2-1B2	RH	Laparoscopy
Yan, 2012 [62]	China	Retrospective cohort	148	1B1	RH and PLND	Laparoscopy
Ma, 2013 [97]	China	Retrospective cohort	50	1B2-2A	RH and PLND	Laparotomy
Mahawerawat, 2013 [63]	Thailand	Retrospective cohort	58	1A2	RH and PLND	Laparotomy
Chai, 2014 [64]	China	Retrospective cohort	438	2B	RH and PLND	Laparotomy
Favero, 2014 [65]	Brazil	Prospective cohort	33	1B2-2B	Extra-fascial hysterectomy	Laparoscopy
Li, 2014 [66]	China	Retrospective cohort	134	1B1-2A2	RH and PLND	Laparoscopy, laparotomy
Makowski, 2014 [67]	Poland	Retrospective cohort	73	1A2-2A1	RH	Laparotomy
Zhang, 2014 [68]	China	Prospective cohort	126	1B1	RH and PLND	Laparotomy
Chen, 2015[98]	China	Retrospective cohort	137	1B-2A	RH	Laparotomy
Durdevic, 2015 [69]	Serbia	Retrospective cohort	175	1B-2B	RH	Laparotomy
Xie, 2015 [70]	China	Retrospective cohort	86	1B1-2A1	RH	Laparoscopy
Yang, 2015 [71]	China	Retrospective cohort	403	1A1-2B	RH and PLND	Laparoscopy
Yang, 2105 [72]	China	Retrospective cohort	120	1B2-2B	RH and PLND + aortic LND	Laparotomy
Gong, 2016 [73]	China	Retrospective cohort	800	1B2-2B	RH	Laparotomy
Liu, 2016 [74]	China	Prospective cohort	120	1B2-2A2	RH	Laparoscopy
Wu, 2016 [75]	China	Retrospective cohort	839	1B1-2A2	RH	Laparotomy
Yang, 2016 [76]	China	Prospective cohort	76	1B1-2A2	RH	Laparoscopy

* RH = radical hysterectomy, PLND = pelvic lymph node dissection, LND = lymph node dissection

** Not reported as retrospective or prospective

Sixty-six separate complications in all studies (high and low quality) were reported on (the full list is available in S1 File).

### Blood transfusion

**Fig 5:**
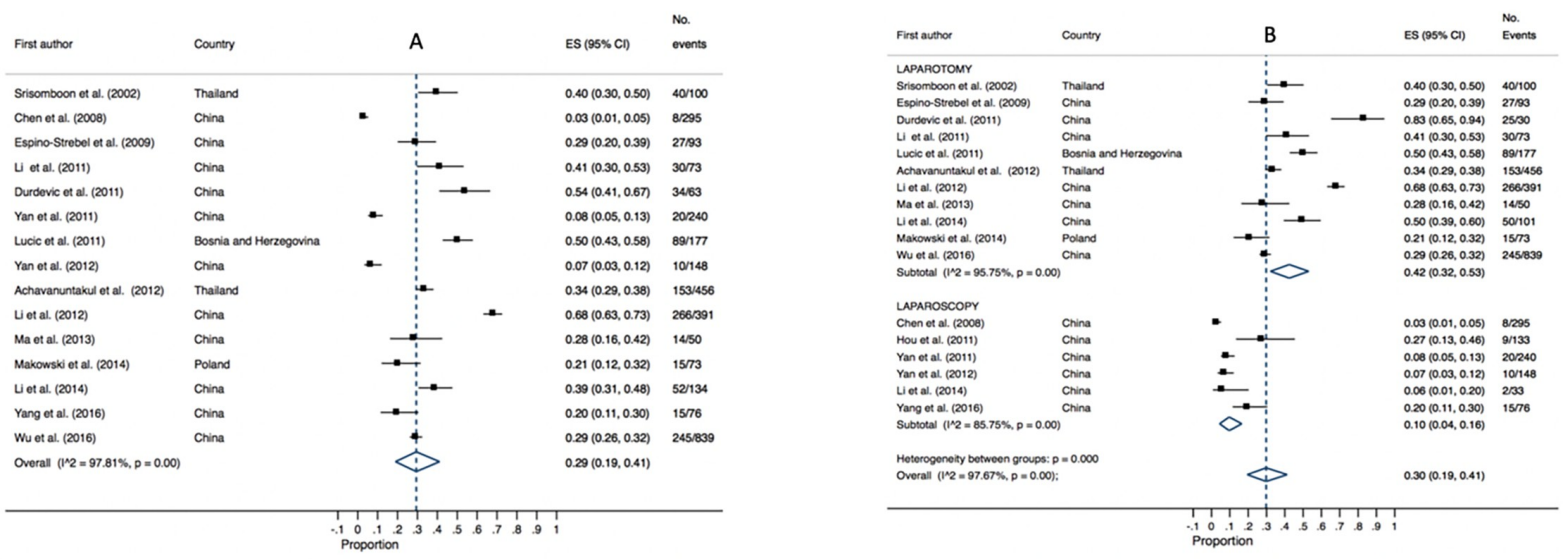
Annotated Forrest plot of overall prevalence of blood transfusion (A) and prevalence by surgical mode (B).

The need for blood transfusion was reported in fifteen studies (3,108 patients) with a pooled estimate of 29% (95% CI 0.19–0.41, P = 0.00, I^2^ = 97.81). Pooled estimates varied greatly between laparotomy cases (42%, 95% CI 0.32–0.53, I^2^ = 95.75, P = 0.00) and laparoscopic cases (10%, 95% CI 0.04–0.16, P = 0.00, I^2^ = 85.75). High heterogeneity was present for both analyses (refer to Fig 5).

### Nerve injury

Four studies (995 patients) reported nerve injury as an intraoperative complication. The overall pooled estimated was 1% (95% CI 0.00–0.03, I^2^ 77.80, P = 0.00). Pooled estimates were higher for laparotomy cases (3%, 95% CI 0.05–0.14) than for laparoscopic cases (0.3%, 95% CI 0.00–0.10). Overall high heterogeneity was identified for both analyses (I^2^ 77.8, P = 0.00).

### Bowel injury

Six studies (1,957 patients) reported bowel injury, with a pooled estimate of 0.5% (95% CI 0.01–0.01, I^2^ = 0.00, P = 0.77). Pooled estimates were slightly higher for laparotomy cases (0.7%, 95% CI 0.01–0.01) than in laparoscopic cases (0.4%, 95% CI 0.00–0.01). Low heterogeneity was identified for both analyses.

### Bladder injury

**Fig 6:**
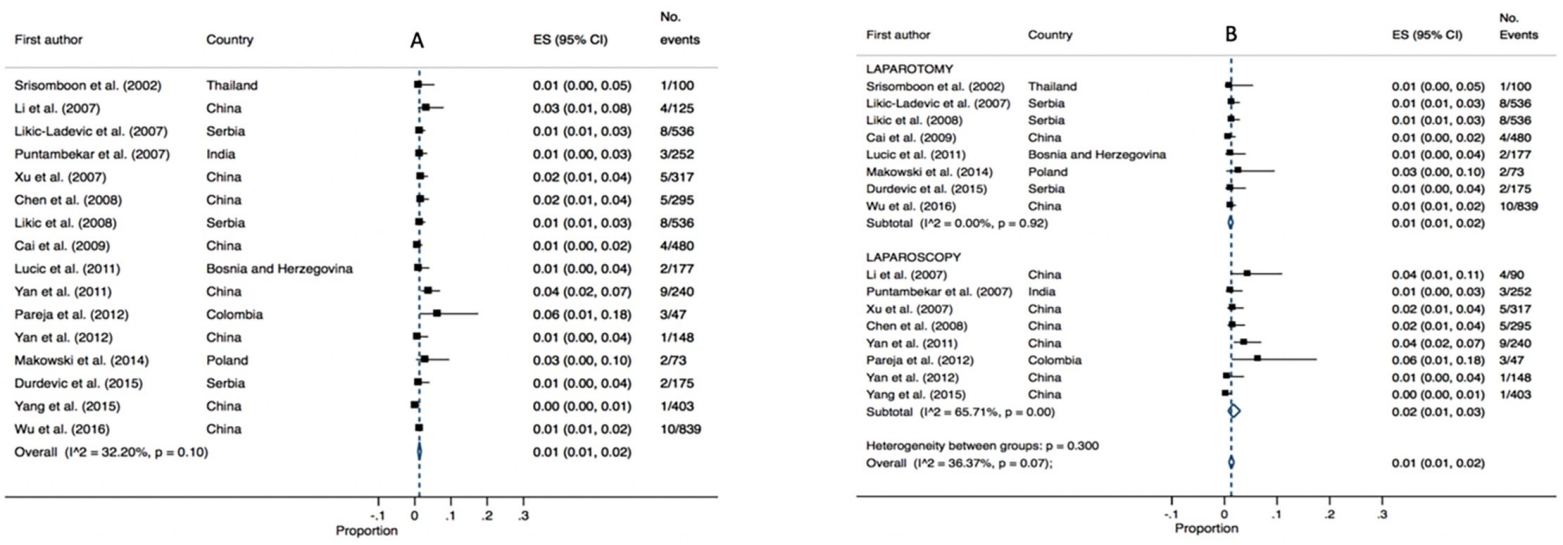
Annotated Forrest plot of overall prevalence of bladder injury (A) and prevalence by surgical mode (B).

Bladder injury was reported in sixteen studies (4,643 patients) and had a pooled estimate of 1% (95% CI 0.01–0.02, P = 0.10, I^2^ = 32.2). Pooled estimates varied slightly between laparotomy cases 1% (95% CI 0.01–0.02, I^2 =^ 0.00, P = 0.92) and laparoscopic cases (2%, 95% CI 0.01–0.02, P = 0.07, I^2^ 36.67). Low heterogeneity was identified for laparotomy and moderate heterogeneity for laparoscopy (refer to Fig 6).

### Ureteric injury

**Fig 7:**
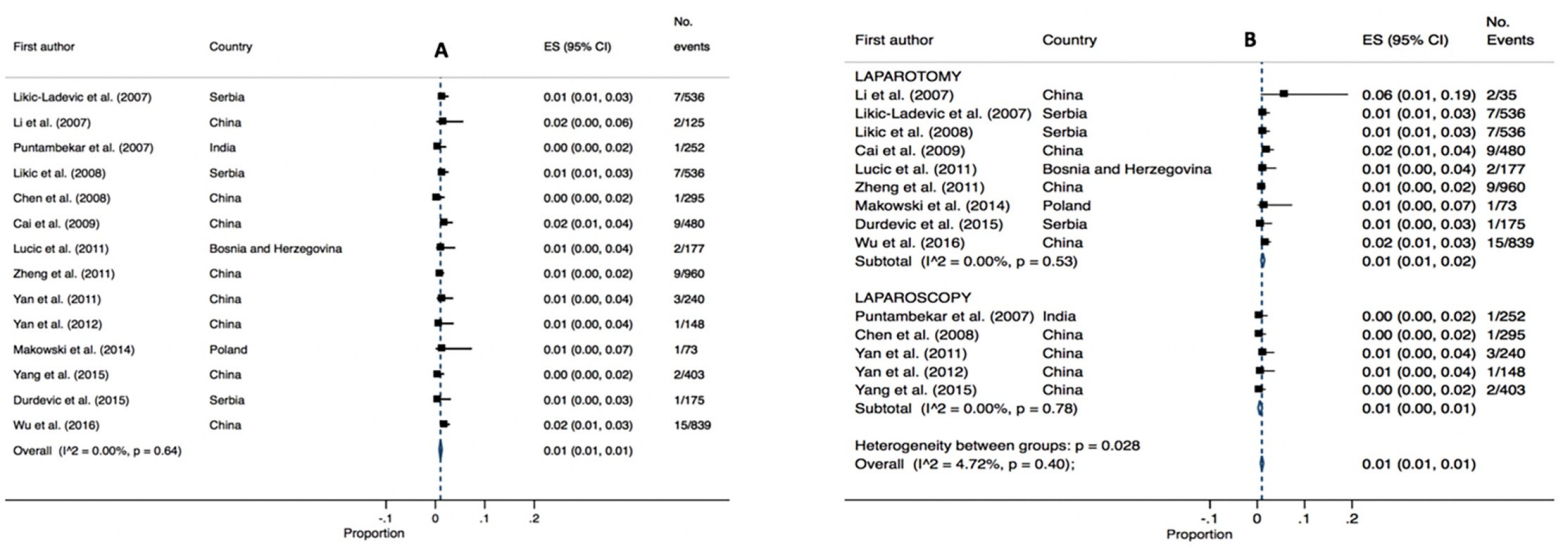
Annotated Forrest plot of overall prevalence of ureteric injury (A) and prevalence by surgical mode (B).

Fourteen studies (3,063 patients) reported ureteric injury, with a pooled estimate of 1% (95% CI 0.01–0.01, I^2^ 0.00, P = 0.64). Pooled estimates were equivalent for laparotomy cases 1% (95% CI 0.01–0.01, I^2^ 0.00, P = 0.53) and laparoscopic cases (1%, 95% CI 0.00–0.01, I^2^ 0.00, P = 0.78). The included studies had low heterogeneity in both analyses (refer to Fig 7).

### Vascular injury

**Fig 8:**
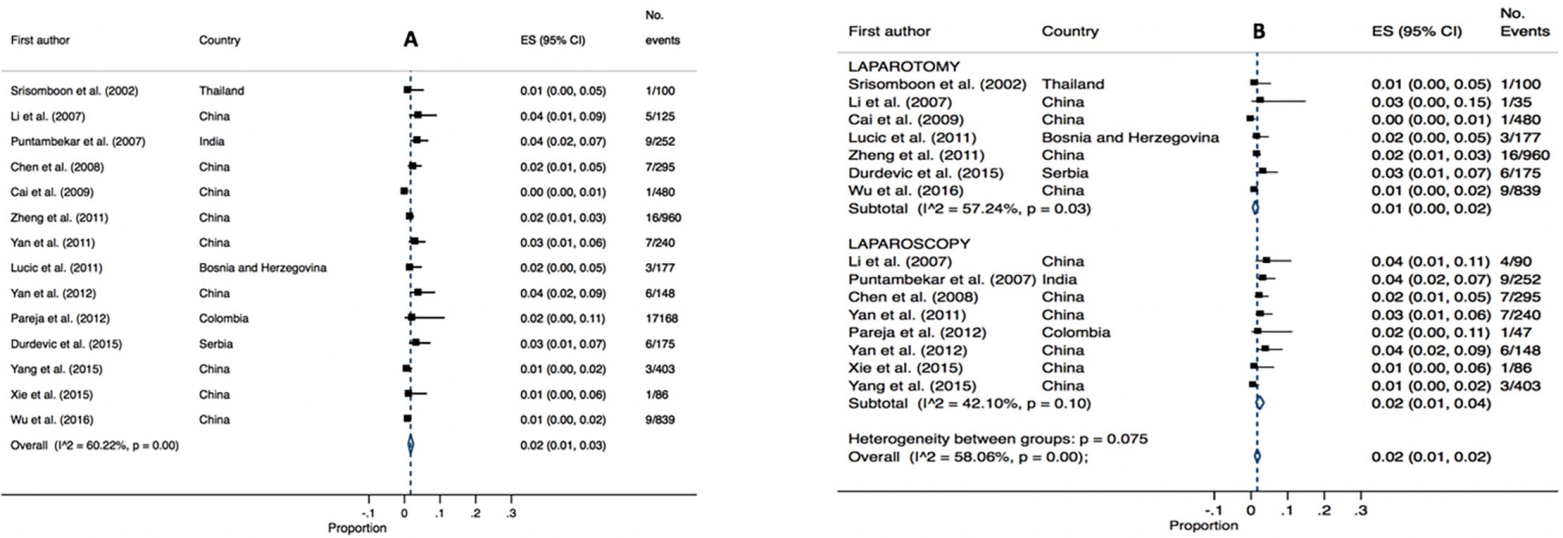
Annotated Forrest plot of overall prevalence of vascular injury (A) and prevalence by surgical mode (B).

Fourteen studies reported vascular injury (2,758 patients) and had a pooled estimate of 2% (95% CI 0.01–0.03, I^2^ 60.22, P = 0.00). Pooled estimates were lower for laparotomy cases 1% (95% CI 0.01–0.04, I^2^ 57.24, P = 0.03) than for laparoscopic cases 2% (95% CI 0.01–0.04, I^2^ 42.10, P = 0.10). Overall high heterogeneity was identified for both analyses (I^2^ 58.06, P = 0.00) (refer to Fig 8).

### Fistula

**Fig 9:**
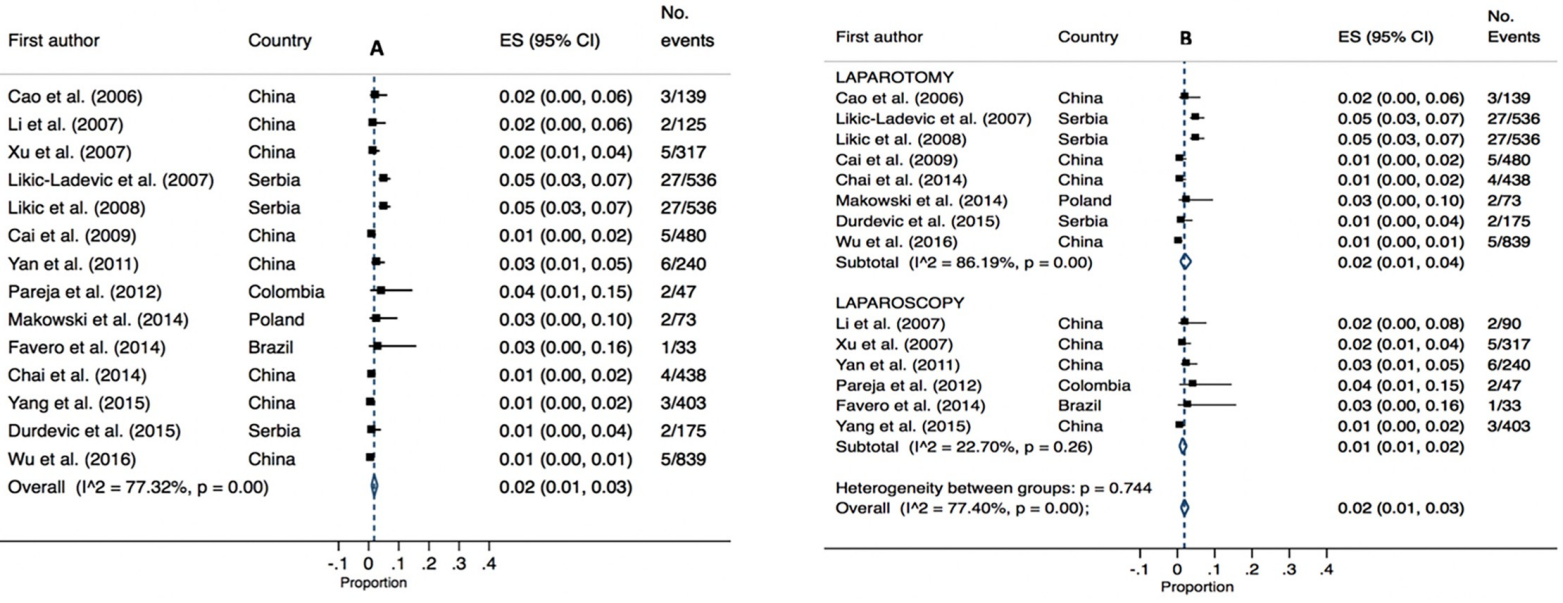
Annotated Forrest plot of overall prevalence of fistula injury (A) and prevalence by surgical mode (B).

Fourteen studies (4,429 patients) reported fistula as a surgical complication. Fistula was defined by the study authors and not necessarily definitive as to type (e.g. vesicovaginal or rectovaginal). The overall pooled estimated was 2% (95% CI 0.01–0.03, I^2^ = 77.32, P = 0.00,). Pooled estimates were comparable between laparotomy cases (2%, 95% CI 0.01–0.04, P = 0.00, I^2 =^ 86.19) and laparoscopic cases (1%, 95% CI 0.01–0.01, I^2^ = 22.70, P = 0.26). Overall high heterogeneity was identified for laparotomy and moderate heterogeneity for laparoscopy (refer to Fig 9).

### Conversion to open surgery

There were three studies (1015 patients) that reported the requirement to convert to open surgery. The overall pooled estimate was 1% (95% CI 0.00–0.02, I^2^ = 21.29, P = 0.28) and the included studies showed moderate heterogeneity.

### Thromboembolic events

Pulmonary embolism was reported in five studies (that included 1,270 patients) as a postoperative complication. The pooled estimate was 0.4% (95% CI 0.00–0.01, I^2^ 26.69, P = 0.25). Seven studies reported on all thromboembolic events (that included 1,501 patients) and had a pooled estimate of 1% (95% CI 0.01–0.02, I^2^ 0.00, P = 0.73). Pooled estimates were comparable for RH (laparotomy) cases (1%, 95% CI 0.00–0.02) vs. RH (laparoscopic) cases (1%, 95% CI 0.00–0.05). Overall the included studies showed low heterogeneity for both analyses (I^2^ 0.00, P = 0.73).

### Infectious morbidity

**Fig 10:**
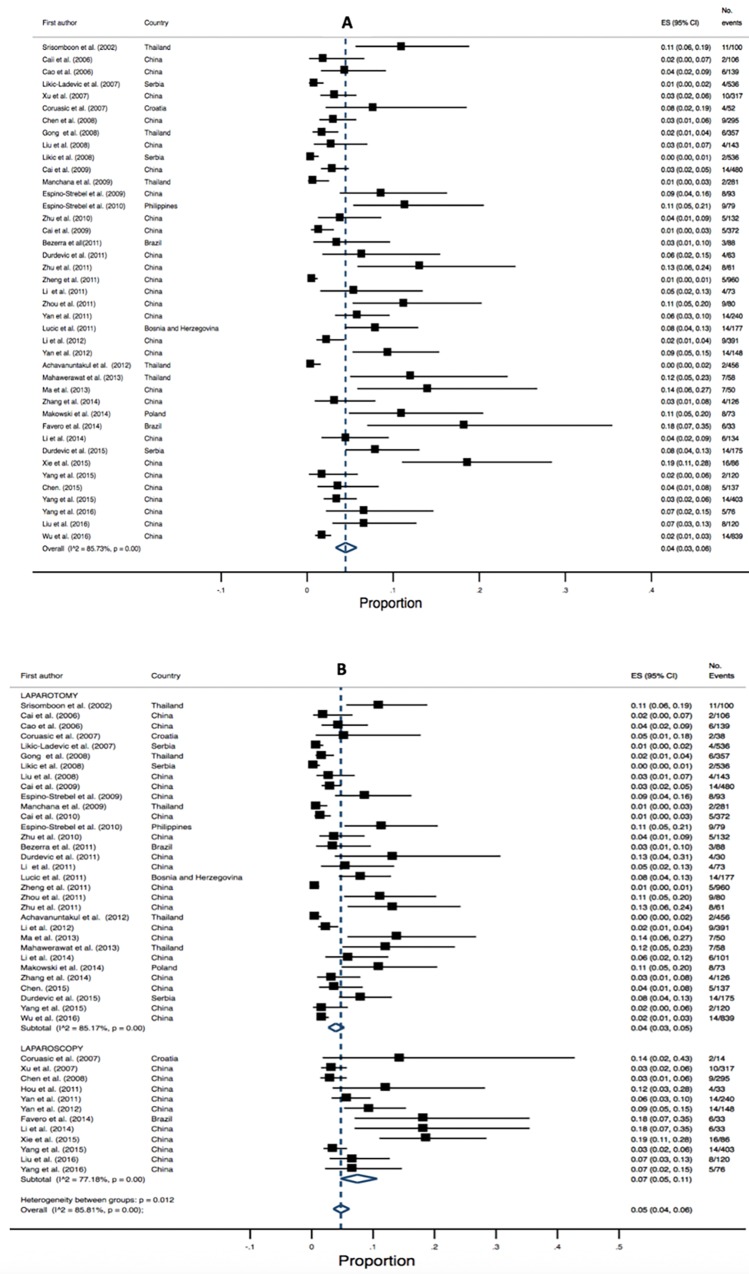
Annotated Forrest plot of overall prevalence of infection (A) and prevalence by surgical mode (B).

There were twenty studies (3,826 patients) that reported infectious morbidity as a postoperative complication. The overall pooled estimated was 8% (95% CI 0.04–0.12, I^2^ 95.72, P = 0.00). Pooled estimates were higher for RH (laparotomy) cases 9% (95% CI 0.05–0.14, I^2 =^ 95.82, P = 0.00) vs. RH (laparoscopic) cases 5% (95% CI 0.00–0.12, I^2^ 88.88, P = 0.00). High heterogeneity was identified for both analyses (refer to Fig 10).

### Assessment of complications excluding studies from China

For the complications of blood transfusion, bladder injury, ureteric injury, vascular injury and fistula, there was less than 0.08 difference in the random pooled estimates when studies from China were excluded. (S2 Table).

### Progression free survival (PFS) and overall survival (OS)

The 5-year PFS was reported for 4,731 patients from 17 studies. Overall PFS was 81.15%, 83% for laparotomy (n = 3,917; 95% CI:0.75–0.89) and 84% for laparoscopy (n = 664; 95% CI:0.81–0.87). The 5-year OS was reported for 5130 patients from 16 studies. OS was 83.5%, 85% for laparotomy cases (n = 4,466; 95% CI: 0.78–0.91) and 80% for laparoscopy (n = 664; 95% CI:0.72–0.88).

### Assessment of publication bias

There was no apparent publication bias for all complications included in the meta-analysis. Funnel plots and p values of Egger’s test for each complication are shown in S2 File

## Discussion

To our knowledge, this is the first systematic review and meta-analysis of morbidity after primary surgical management of cervical cancer specifically in LMIC, although only data from middle-income countries (MIC) were included in the meta-analysis. The incidence of complications in this meta-analysis is largely comparable to those reported in high-income countries (HIC). Blood transfusions are reported in HIC at up to 25% for minimally invasive surgical approaches for cervical cancer and up to 75% for laparotomy[80], and so our findings of 10% and 42% respectively are consistent with published studies that have been conducted in well resourced settings. This may however also reflect differing resources and thresholds for transfusions in LMIC. In HIC 1.2% and 2.8% of women develop fistula (ureterovaginal and vesicovaginal) and pulmonary embolism respectively[81] following surgery for cervical cancer. Ureteric injuries following laparotomy and radical hysterectomy for cervical cancer were reported to occur in 2.48% of cases in a review of nearly 400,000 hysterectomies in the United Kingdom (UK)[82]. The reported incidence of complications is similar or lower in this meta-analysis and may represent publication bias. However, our findings suggest that, morbidity following surgery for cervical cancer in MIC appears to compare favourably to the reported incidence in HIC.

The comparative survival between laparoscopy and laparotomy cases in this review is clearly different to that of a recently published retrospective cohort study [83] and prospective RCT in HIC [84]. However, our data is neither registry based nor largely prospective so comparisons should be made with caution. It is reassuring that the risks of complications reported in this meta-analysis are comparable to HICs. Accepting that some settings within LMIC have individual survival rates more comparable to HIC[85], overall long-term outcomes do not compare favourably for cervical cancer patients in LMIC where overall 5-year survival rate is 50%, which is markedly different to HIC, which report survival rates up to 80%. Reasons for this difference may include the stages of tumour (e.g. 2B), being offered operative care which may not happen in HIC, high rates of HIV positivity in these settings in women who are younger at diagnosis than non-HIV positive women, although in many settings their outcomes appear comparable[86]. Equally, surgeons are frequently the mainstay of treatment for women in LMIC and the lack of adjuvant treatment likely impacts on outcomes[3, 6].

The strengths of the current study include the rigorous inclusion criteria and a search that was not limited by language. Moreover, the broad search criteria were not limited to LMIC (which are not necessarily linked in MeSH terms or titles and abstracts) and so this increased the likelihood of capturing studies reporting on outcomes in LMIC. A large number of both articles and patients were included in the meta-analysis with a surgical treatment period since 2000, which is likely reflective of current practice. Each complication has a large denominator, although we accept that the definition of morbidity (in both high income and LMIC studies) is not necessarily standardised, and subject to variations in regional practices and definitions[87]. However, we have only included high quality studies in the meta-analysis, adding to its robustness.

There are several limitations to this study. As the main body of data are derived from retrospective studies with high heterogeneity, a random-effects model was applied to decrease the possibility of overestimating surgical morbidity. Nevertheless, the retrospective and unblinded study designs of the majority of included studies may introduce biases that cannot be evaluated. Furthermore, there is variability in surgical approaches, including lymphadenectomy, that potentially affect the meta-analysis; however this is reflective of current surgical practice, and the issue is similar to that encountered in other meta-analyses of surgical complications[88].

Operative morbidity could also be influenced by neoadjuvant chemotherapy. A recent randomized trial from India reported inferior disease-free survival (DFS) with neoadjuvant-chemotherapy followed by surgery compared to concurrent chemoradiation. This study has also reported operative morbidity. Patients randomised to neoadjuvant chemotherapy had a significantly higher rate of grade 3 or 4 thrombocytopenia compared to those treated by concurrent chemoradiation, but there was no significant difference between the two groups in grade 3 or 4 gastrointestinal and bladder toxicities. In the neoadjuvant chemotherapy plus surgery group, perioperative hemorrhage with 1000 mL blood loss occurred in 7.9% of patients[89]. In view of the superior DFS with chemoradiation it is conceivable that the neoadjuvant modality might decline in the future. Due to the heterogeneity of the regimens of the included studies, it was not possible to analyse the impact of neoadjuvant and adjuvant therapies on surgical morbidity in this meta-analysis.

Only one high quality study[65] reported on simple hysterectomy rather than radical hysterectomy. However, this study included only 33 patients and so is unlikely to impact on the outcome of the meta-analyses. Equally patient co-morbidities that confound complication results are not reported. No high-quality studies from LICs were included and there is little representation from Africa in this analysis (2 studies from this region that met inclusion criteria were excluded at the quality assessment stage) and furthermore, data from China represents 65% of the included studies. This may account for the reported incidences of surgical complications in the current meta-analysis, which are comparable to those in high-income countries, as many parts of China are increasingly becoming higher income settings[90]. However, the similar pooled estimates for reported complications in our sensitivity analysis that excluded studies from China do not support this. It is also plausible that the surgical case load and experience in caring for cervical cancer cases in LMIC mitigates the limitations in these settings and results in comparable surgical complications with HIC. An important limitation is that included data were not population or registry based. This may explain the relatively high progression free and overall survival reported in our study, which is markedly different to that reported by Allemani and colleagues, with 5 year OS in LMIC of around 50% [91], based on population registry data that included patients with all stages of cervical cancer. Finally, it was not possible to stratify surgical complications by stage of disease or timing of the complication (e.g. immediately post-operative or delayed complication) as this was infrequently reported in the included studies.

Given these limitations, there is a clear lack of globally comprehensive and representative data on surgical outcomes in LMIC[8, 92], which, by virtue of inadequate assessment of deficiencies where resources can be targeted, and by limiting knowledge on the natural history and variations in cancer outcomes in these settings, worsens cancer care[3]. Data are particularly lacking from LICs. Developing registries to meet this gap is critical in addressing the global burden of non-communicable diseases including cancer[93], particularly with increasing access to adjuvant therapies in these settings. Inclusive access to the most basic cervical cancer management is lacking in LMIC and system strengthening is critical to changing this[94]. The Lancet Commission on Global Surgery clearly outlines that scale-up of universal access to safe surgical care includes the need for strong system development grounded in data collection, including surgical indicators, such that system issues and deficiencies can be identified and changes advocated for. If we are to meet these goals then, similar to global efforts to count every newborn[95], the oncology community urgently needs to work with LMIC policy makers to ensure longitudinal data collection and the counting of every cancer case[91, 92].

## Conclusion

This is the first systematic review and meta-analysis of surgical morbidity in cervical cancer in LMIC and provides a benchmark for future health services research and policy implementation. Surgery is the cornerstone of management in early stage cervical cancer and is likely to remain a major treatment modality in LMIC for many years; we must as a global oncology community mobilise to ensure the foundations for health advocacy and program development are in place, including surgical morbidity registries.

## Supporting information

S1 Table Complete search strategy. (DOCX)

S2 Table Sensitivity analysis for surgical complications reported in the meta-analysis. (DOCX)

S1 File All reported complications in included studies. (DOCX)

S2 File Funnel plots reporting surgical complications for all studies included in the meta-analysis. (DOCX)

S3 File PRISMA checklist. (PDF)

S4 File Surgical complications in high quality studies. (XLSX)

S5 File Surgical complications (laparoscopy and laparotomy) in high quality studies. (XLSX)

## References

[1] Bray F, Ferlay J, Soerjomataram I, Siegel RL, Torre LA, Jemal A. Global cancer statistics 2018: GLOBOCAN estimates of incidence and mortality worldwide for 36 cancers in 185 countries. CA: a cancer journal for clinicians. 2018. 10.3322/caac.21492 30207593

[2] Arbyn M, Castellsague X, de Sanjose S, Bruni L, Saraiya M, Bray F, et al Worldwide burden of cervical cancer in 2008. Ann Oncol. 2011;22(12):2675–86. Epub 2011/04/08. 10.1093/annonc/mdr015 . 21471563

[3] Kingham TP, Alatise OI, Vanderpuye V, Casper C, Abantanga FA, Kamara TB, et al Treatment of cancer in sub-Saharan Africa. The Lancet Oncology. 2013;14(4):e158–e67. 10.1016/S1470-2045(12)70472-2 23561747

[4] Gelband H, Sankaranarayanan R, Gauvreau CL, Horton S, Anderson BO, Bray F, et al Costs, affordability, and feasibility of an essential package of cancer control interventions in low-income and middle-income countries: key messages from Disease Control Priorities. The Lancet. 2016;387(10033):2133–44. 10.1016/S0140-6736(15)00755-2 26578033

[5] Small W, Bacon MA, Bajaj A, Chuang LT, Fisher BJ, Harkenrider MM, et al Cervical cancer: A global health crisis. Cancer. 2017. 10.1002/cncr.30667 28464289

[6] Coleman JS, Cespedes MS, Cu-Uvin S, Kosgei RJ, Maloba M, Anderson J, et al An Insight Into Cervical Cancer Screening and Treatment Capacity in Sub Saharan Africa. J Low Genit Tract Dis. 2016;20(1):31–7. Epub 2015/11/19. 10.1097/LGT.0000000000000165 26579842 PMC4691409

[7] Kingham TP, Alatise OI, Vanderpuye V, Casper C, Abantanga FA, Kamara TB, et al Treatment of cancer in sub-Saharan Africa. Lancet Oncol. 2013;14(4):e158–67. Epub 2013/04/09. 10.1016/S1470-2045(12)70472-2 . 23561747

[8] Biccard BM, Madiba TE, Kluyts H-L, Munlemvo DM, Madzimbamuto FD, Basenero A, et al Perioperative patient outcomes in the African Surgical Outcomes Study: a 7-day prospective observational cohort study. The Lancet. 2018.

[9] Samarasekera U, Horton R. Women’s cancers: shining a light on a neglected health inequity. The Lancet. 2017;389(10071):771–3. 10.1016/S0140-6736(16)31798-6 27814968

[10] Clayton RD. Hysterectomy. Best Practice & Research Clinical Obstetrics & Gynaecology. 2006;20(1):73–87. 16275095 10.1016/j.bpobgyn.2005.09.007

[11] Sandadi S, Tanner EJ, Khoury-Collado F, Kostolias A, Makker V, Chi DS, et al Radical surgery with individualized postoperative radiation for stage IB cervical cancer: oncologic outcomes and severe complications. Int J Gynecol Cancer. 2013;23(3):553–8. Epub 2013/02/09. 10.1097/IGC.0b013e3182849d53 . 23392402

[12] Lacorre A, Merlot B, Garabedian C, Narducci F, Chereau E, Resbeut M, et al Early stage cervical cancer: Brachytherapy followed by type a hysterectomy versus type B radical hysterectomy alone, a retrospective evaluation. European Journal of Surgical Oncology (EJSO). 2016;42(3):376–82. 26725307 10.1016/j.ejso.2015.12.003

[13] Reade CJ, Eiriksson LR, Covens A. Surgery for early stage cervical cancer: how radical should it be? Gynecol Oncol. 2013;131(1):222–30. Epub 2013/07/19. 10.1016/j.ygyno.2013.07.078 . 23863357

[14] Alexander-Sefre F, Chee N, Spencer C, Menon U, Shepherd JH. Surgical morbidity associated with radical trachelectomy and radical hysterectomy. Gynecol Oncol. 2006;101(3):450–4. Epub 2005/12/14. 10.1016/j.ygyno.2005.11.007 . 16343604

[15] Moher D, Liberati A, Tetzlaff J, Altman DG, Group P. Preferred reporting items for systematic reviews and meta-analyses: the PRISMA statement. PLoS medicine. 2009;6(7):e1000097 10.1371/journal.pmed.1000097 19621072 PMC2707599

[16] World Bank Group. The World Bank Data [Internet] 2018 [cited 2018 15th May]. Available from: https://data.worldbank.org/income-level/low-and-middle-income.

[17] World Bank Group. Where are your data on Taiwan? 2018 [cited 2018 23rd May]. Available from: https://datahelpdesk.worldbank.org/knowledgebase/articles/114933-where-are-your-data-on-taiwan.

[18] Wells G, Shea B, O’connell D, Peterson J, Welch V, Losos M, et al The Newcastle-Ottawa Scale (NOS) for assessing the quality of nonrandomised studies in meta-analyses. 2000.

[19] Pratt JJ, Niedle PS, Vogel JP, Oladapo OT, Bohren M, Tunçalp Ö, et al Alternative regimens of magnesium sulfate for treatment of preeclampsia and eclampsia: a systematic review of non‐randomized studies. Acta obstetricia et gynecologica Scandinavica. 2016;95(2):144–56. 10.1111/aogs.12807 26485229

[20] Long Q, Allanson ER, Pontre J, Tunçalp Ö, Hofmeyr GJ, Gülmezoglu AM. Onsite midwife-led birth units (OMBUs) for care around the time of childbirth: a systematic review. BMJ Global Health. 2016;1(2):e000096 10.1136/bmjgh-2016-000096 28588944 PMC5321346

[21] Editors PM. Observational studies: getting clear about transparency. PLoS Med. 2014;11(8):e1001711 10.1371/journal.pmed.1001711 25158064 PMC4144975

[22] Higgins JP. Green S. Cochrane handbook for systematic reviews of interventions version 5.1. 0. The cochrane collaboration. 2011;5(0).

[23] Lo CK-L, Mertz D, Loeb M. Newcastle-Ottawa Scale: comparing reviewers’ to authors’ assessments. BMC medical research methodology. 2014;14(1):45. 24690082 10.1186/1471-2288-14-45 PMC4021422

[24] Newcombe RG. Two‐sided confidence intervals for the single proportion: comparison of seven methods. Statistics in medicine. 1998;17(8):857–72. 9595616 10.1002/(sici)1097-0258(19980430)17:8<857::aid-sim777>3.0.co;2-e

[25] Harris R, Bradburn M, Deeks J, Harbord R, Altman D, Sterne J. Metan: fixed-and random-effects meta-analysis. Stata journal. 2008;8(1):3.

[26] Higgins JP, Thompson SG, Deeks JJ, Altman DG. Measuring inconsistency in meta-analyses. BMJ: British Medical Journal. 2003;327(7414):557 10.1136/bmj.327.7414.557 12958120 PMC192859

[27] Higgins JP, Green S. Cochrane handbook for systematic reviews of interventions: John Wiley & Sons; 2011.

[28] Light RJ, Singer JD, Willett JB. The visual presentation and interpretation of meta-analyses The handbook of research synthesis. New York, NY, US: Russell Sage Foundation; 1994 p. 439–53.

[29] Sutton AJ, Abrams KR, Jones DR, Jones DR, Sheldon TA, Song F. Methods for meta-analysis in medical research. 2000.

[30] Egger M, Smith GD, Schneider M, Minder C. Bias in meta-analysis detected by a simple, graphical test. Bmj. 1997;315(7109):629–34. 10.1136/bmj.315.7109.629 9310563 PMC2127453

[31] Nyaga VN, Arbyn M, Aerts M. Metaprop: a Stata command to perform meta-analysis of binomial data. Arch Public Health. 2014;72(1):39 Epub 2014/01/01. 10.1186/2049-3258-72-39 25810908 PMC4373114

[32] Cao D, Yang J, Wu X, Chen Y, Li L, Liu K, et al Comparisons of vaginal and abdominal radical trachelectomy for early-stage cervical cancer: preliminary results of a multi-center research in China. British journal of cancer. 2013;109(11):2778 10.1038/bjc.2013.656 24169350 PMC3844910

[33] Li J, Wu X, Li X, Ju X. Abdominal radical trachelectomy: Is it safe for IB1 cervical cancer with tumors≥ 2 cm? Gynecologic oncology. 2013;131(1):87–92. 10.1016/j.ygyno.2013.07.079 23872192

[34] Srisomboon J, Phongnarisorn C, Suprasert P, Cheewakriangkrai C, Siriaree S, Charoenkwan K. A prospective randomized study comparing retroperitoneal drainage with no drainage and no peritonization following radical hysterectomy and pelvic lymphadenectomy for invasive cervical cancer. Journal of Obstetrics & Gynaecology Research. 2002;28(3):149–53. 12214830 10.1046/j.1341-8076.2002.00027.x

[35] Cai HB, Chen HZ, Yin HH. Randomized study of preoperative chemotherapy versus primary surgery for stage IB cervical cancer. Journal of Obstetrics & Gynaecology Research. 2006;32(3):315–23. 16764623 10.1111/j.1447-0756.2006.00404.x

[36] Cao L, Li X, Zhang Y, Li X. Surgical pattern of radical hysterectomy and pelvic lymph node dissection for patients with cervical cancer. Zhong nan da xue xue bao Yi xue ban = Journal of Central South University Medical sciences. 2006;31(4):588–90. 16951525

[37] Ćorušić A, Barišić D, Plavec A, Planinić P, Škrgatić L, Vujić G, et al Laporovaginal surgery in cervical cancer: a Croatian experience. Collegium antropologicum. 2007;31(2):147–54. 17598518

[38] Li G, Yan X, Shang H, Wang G, Chen L, Han Y. A comparison of laparoscopic radical hysterectomy and pelvic lymphadenectomy and laparotomy in the treatment of Ib-IIa cervical cancer. Gynecologic oncology. 2007;105(1):176–80. 10.1016/j.ygyno.2006.11.011 17197013

[39] Likić-Lađević I, Kadija S, Lađević N, Stefanović A, Argirović R, Petković S, et al Urological complications after radical hysterectomy: incidence rates and predisposing factors. Vojnosanitetski pregled. 2007;64(6):381–4. 17687941 10.2298/vsp0706381l

[40] Puntambekar SP, Palep RJ, Puntambekar SS, Wagh GN, Patil AM, Rayate NV, et al Laparoscopic total radical hysterectomy by the Pune technique: our experience of 248 cases. Journal of minimally invasive gynecology. 2007;14(6):682–9. 10.1016/j.jmig.2007.05.007 17980327

[41] Xu H, Chen Y, Li Y, Zhang Q, Wang D, Liang Z. Complications of laparoscopic radical hysterectomy and lymphadenectomy for invasive cervical cancer: experience based on 317 procedures. Surgical endoscopy. 2007;21(6):960 10.1007/s00464-006-9129-0 17287919

[42] Chen Y, Xu H, Li Y, Wang D, Li J, Yuan J, et al The outcome of laparoscopic radical hysterectomy and lymphadenectomy for cervical cancer: a prospective analysis of 295 patients. Annals of Surgical Oncology. 2008;15(10):2847–55. 10.1245/s10434-008-0063-3 18649105

[43] Kietpeerakool C, Lattiwongsakorn W, Srisomboon J. Incidence and predictors of febrile morbidity after radical hysterectomy and pelvic lymphadenectomy for early stage cervical cancer patients. Asian Pac J Cancer Prev. 2008;9(2):213–6. 18712961

[44] Likic IS, Kadija S, Ladjevic NG, Stefanovic A, Jeremic K, Petkovic S, et al Analysis of urologic complications after radical hysterectomy. American Journal of Obstetrics & Gynecology. 2008;199(6):644. e1–. e3. 10.1016/j.ajog.2008.06.034 18722569

[45] Cai H-B, Chen H-Z, Zhou Y-F, Lie D-M, Hou H-Y. Class II radical hysterectomy in low-risk IB squamous cell carcinoma of cervix: a safe and effective option. International Journal of Gynecological Cancer. 2009;19(1):46–9. 10.1111/IGC.0b013e318197f847 19258940

[46] Ju X, Li Z, Yang H, Wu X. Nerve-sparing radical hysterectomy and radical hysterectomy: a retrospective study. Zhonghua fu chan ke za zhi. 2009;44(8):605–9. 20003790

[47] Manchana T, Sirisabya N, Lertkhachonsuk R, Worasethsin P, Khemapech N, Sittisomwong T, et al Long term complications after radical hysterectomy with pelvic lymphadenectomy. Medical journal of the Medical Association of Thailand. 2009;92(4):451. 19374292

[48] Cai H, Chen H, Zhang F, Nie D, Xiong Y, Liu L. Clinical report of the modified Piver class III hysterectomy on invasive cervical cancer. Zhonghua fu chan ke za zhi. 2010;45(7):511–4. 21029603

[49] Espino-Strebel EE, Luna JTP, Domingo EJ. A comparison of the feasibility and safety of nerve-sparing radical hysterectomy with the conventional radical hysterectomy. International Journal of Gynecological Cancer. 2010;20(7):1274–83. 21495251 10.1111/igc.0b013e3181f165f2

[50] Zhu J, Luo J, Zhang W, Cao Y, Huang Y. The effect of preoperative interventional therapy on short-term and long-term therapeutic results of radical surgery for early cervical cancer. Journal of Interventional Radiology. 2010;19(1):28–31.

[51] Bezerra ALR, Martins MR, Bezerra SMMDS, Figueiroa JN, Batista TP. Class II radical hysterectomy for stage I–IIA cervix cancer: Prognostic factors associated to recurrence and survival in a northeast Brazil experience. Journal of Surgical Oncology. 2011;104(3):255–9. 10.1002/jso.21939 21465491

[52] Hou CY, Li XL, Jiang F, Gong RJ, Guo XY, Yao YQ. Comparative evaluation of surgical stress of laparoscopically assisted vaginal radical hysterectomy and lymphadenectomy and laparotomy for early‑stage cervical cancer. Oncology letters. 2011;2(4):747–52. 10.3892/ol.2011.311 22848260 PMC3406333

[53] Li B, Li W, Sun Y, Zhang R, Zhang G, Yu G, et al Nerve plane-sparing radical hysterectomy: a simplified technique of nerve-sparing radical hysterectomy for invasive cervical cancer. Chinese medical journal. 2011;124(12):1807–12. 21740837

[54] Lučić N, Antonić Z, Ećim V, Draganović D, Latinović L. Treatment of cervical cancer in the Republic of Srpska. Medicinski pregled. 2011;64(11–12):588–91. 22369006 10.2298/mpns1112588l

[55] Yan X, Li G, Shang H, Wang G, Han Y, Lin T, et al Twelve-year experience with laparoscopic radical hysterectomy and pelvic lymphadenectomy in cervical cancer. Gynecologic oncology. 2011;120(3):362–7. 10.1016/j.ygyno.2010.11.033 21168904

[56] Zheng M, Huang L, He L, Ding H, Wang HY, Zheng LM. Evaluation of the effects of type II radical hysterectomy in the treatment of 960 patients with stage IB–IIB cervical carcinoma: A retrospective study. Journal of surgical oncology. 2011;103(5):435–41. 10.1002/jso.21800 21400530

[57] ZHOU L-h, WU J, JIANG X-d. Preoperative brachytherapy combined with operation for stageⅠb2~ Ⅱa cervical cancer [J]. Journal of Practical Oncology. 2011;4:014.

[58] Zhu T, Yu A, Shou H, Chen X, Zhu J, Yang Z, et al Feasibility of unilateral or bilateral nerve-sparing radical hysterectomy in patients with cervical cancer and evaluation of the post-surgery recovery of the bladder and rectal function. Zhonghua zhong liu za zhi [Chinese journal of oncology]. 2011;33(1):53–7. 21575466

[59] Achavanuntakul K, Charoenkwan K. Factors affecting operative blood loss from open radical hysterectomy and pelvic lymphadenectomy for early-stage cervical cancer. Archives of Gynecology & Obstetrics. 2012;286(4):1001–5. 22622853 10.1007/s00404-012-2387-2

[60] Li L, Qie MR, Wang XL, Huang J, Zhang Q, Li DQ, et al BiClamp® forceps was significantly superior to conventional suture ligation in radical abdominal hysterectomy: a retrospective cohort study in 391 cases. Archives of Gynecology & Obstetrics. 2012;286(2):457–63. 22456787 10.1007/s00404-012-2275-9

[61] Pareja R, Nick AM, Schmeler KM, Frumovitz M, Soliman PT, Buitrago CA, et al Quality of laparoscopic radical hysterectomy in developing countries: a comparison of surgical and oncologic outcomes between a comprehensive cancer center in the United States and a cancer center in Colombia. Gynecologic oncology. 2012;125(2):326–9. 10.1016/j.ygyno.2012.01.007 22261300 PMC4286382

[62] Yan X, Li G, Shang H, Lin F, Yang X, Zheng F. Outcome and prognostic factors of laparoscopic radical hysterectomy and pelvic lymphadenectomy in 148 patients with stage IB1 cervical cancer. International Journal of Gynecological Cancer. 2012;22(2):286–90. 10.1097/IGC.0b013e318233d549 22146764

[63] Mahawerawat S, Charoenkwan K, Srisomboon J, Khunamornpong S, Suprasert P, Sae-Teng CT. Surgical outcomes of patients with stage IA2 cervical cancer treated with radical hysterectomy. Asian Pacific Journal of Cancer Prevention. 2013;14(9):5375–8. 10.7314/apjcp.2013.14.9.5375 24175829

[64] Chai Y, Wang T, Wang J, Yang Y, Gao Y, Gao J, et al Radical hysterectomy with adjuvant radiotherapy versus radical radiotherapy for FIGO stage IIB cervical cancer. BMC cancer. 2014;14(1):63. 24495453 10.1186/1471-2407-14-63 PMC3918172

[65] Favero G, Pierobon J, Genta ML, Araújo MP, Miglino G, Diz MDCP, et al Laparoscopic extrafascial hysterectomy (completion surgery) after primary chemoradiation in patients with locally advanced cervical cancer: technical aspects and operative outcomes. International Journal of Gynecological Cancer. 2014;24(3):608–14. 10.1097/IGC.0000000000000067 24503812

[66] Li B, Yao H, Zuo J, Yang Y, Wang W, Zhang G, et al Application of laparoscopy in the modified nerve plane-sparing radical hysterectomy of cervical cancer. Zhonghua zhong liu za zhi [Chinese journal of oncology]. 2014;36(1):63–8. 24685090

[67] Makowski M, Nowak M, Szpakowski M, Władziński J, Serwach-Nowińska A, Janas Ł, et al Classical radical hysterectomy and nerve-sparing radical hysterectomy in the treatment of cervical cancer. Przeglad menopauzalny = Menopause review. 2014;13(3):180 10.5114/pm.2014.43822 26327852 PMC4520361

[68] Zhang D, Li J, Ge H, Ju X, Chen X, Tang J, et al Surgical and pathological outcomes of abdominal radical trachelectomy versus hysterectomy for early-stage cervical cancer. International Journal of Gynecological Cancer. 2014;24(7):1312–8. 10.1097/IGC.0000000000000185 24987922

[69] Đurđević S, Stojanović S, Pantelić M, Nikolić D, Basta-Nikolić M, Mocko-Kaćanski M. Radical hysterectomy in surgical treatment of invasive cervical cancer at the department of gynecology and obstetrics in Novi Sad in the period 1993–2013. Medicinski pregled. 2015;68(7–8):227–33. 26591634 10.2298/mpns1508227d

[70] Xie Q, Deng K, Zheng Y, Wang H, Huang X, Liu X. Modified radical vaginal hysterectomy for cervical cancer treatment. European journal of gynaecological oncology. 2015;36(5):554–9. 26513882

[71] Yang L, Cai J, Dong W, Shen Y, Xiong Z, Wang H, et al Laparoscopic radical hysterectomy and pelvic lymphadenectomy can be routinely used for treatment of early-stage cervical cancer: a single-institute experience with 404 patients. Journal of minimally invasive gynecology. 2015;22(2):199–204. 10.1016/j.jmig.2014.09.009 25281840

[72] YANG X-g, Shi Z, LI Z-w, Ge W, Wei L, WEN H-c, et al Neoadjuvant chemotherapy via different approaches for the treatment of cervical carcinoma in young female patients: comparison of the therapeutic effect. Journal of Interventional Radiology. 2015;(4):342–6.

[73] Gong L, Zhang J-W, Yin R-T, Wang P, Liu H, Zheng Y, et al Safety and efficacy of neoadjuvant chemotherapy followed by radical surgery versus radical surgery alone in locally advanced cervical cancer patients. International Journal of Gynecological Cancer. 2016;26(4):722–8. 10.1097/IGC.0000000000000658 26905330

[74] Liu Z, Li X, Tao Y, Li W, Yang Y, Yao Y, et al Clinical efficacy and safety of laparoscopic nerve-sparing radical hysterectomy for locally advanced cervical cancer. International Journal of Surgery. 2016;25:54–8. 10.1016/j.ijsu.2015.11.029 26632655

[75] Wu M-f, Li J, Lu H-w, Wang L-j, Zhang B-z, Lin Z-q. Impact of the care provided by gynecologic oncologists on outcomes of cervical cancer patients treated with radical hysterectomy. OncoTargets & Therapy. 2016;9:1361. 27022291 10.2147/OTT.S99874 PMC4792213

[76] Yang Y, Qin T, Zhang W, Wu Q, Yang A, Xu F. Laparoscopic nerve-sparing radical hysterectomy for bulky cervical cancer (≥ 6 cm) after neoadjuvant chemotherapy: A multicenter prospective cohort study. International Journal of Surgery. 2016;34:35–40. 10.1016/j.ijsu.2016.08.001 27519498

[77] Fuxiang L, Huashu Li, Zhaoxia Mo. Therapeutic effect of preoperative interventional therapy on IB2~IIB cervical cancer. Chinese Oncology Clinical,. 2008;35(20):1165–7.

[78] Yao C. Comparison of curative effect between laparoscopic radical hysterectomy with pelvic nerve and traditional extensive hysterectomy. Journal of Dalian Medical University. 2015;1:49–52.

[79] Yaomei M, Guiling Z, Wei Y, Peisong S, Caiyan L, Fengxia X. Preoperative adjuvant chemotherapy and radiotherapy combined with radical hysterectomy for the treatment of 50 cases of stage I B2-IIA cervical cancer. Chinese Journal of Clinical Oncology. 2013;40(8):471–4.

[80] Corrado G, Vizza E, Legge F, Anchora LP, Sperduti I, Fagotti A, et al Comparison of Different Surgical Approaches for Stage IB1 Cervical Cancer Patients: A Multi-institution Study and a Review of the Literature. International Journal of Gynecological Cancer. 2018;28(5):1020–8. 10.1097/IGC.0000000000001254 29727351

[81] Berek JS, Hacker NF. Berek and Hacker's gynecologic oncology: Lippincott Williams & Wilkins; 2015.

[82] Kiran A, Hilton P, Cromwell D. The risk of ureteric injury associated with hysterectomy: a 10‐year retrospective cohort study. BJOG: An International Journal of Obstetrics & Gynaecology. 2016;123(7):1184–91. 26281794 10.1111/1471-0528.13576

[83] Melamed A, Margul DJ, Chen L, Keating NL, del Carmen MG, Yang J, et al Survival after minimally invasive radical hysterectomy for early-stage cervical cancer. New England Journal of Medicine. 2018. 10.1056/NEJMoa1804923 PMC6464372 30379613

[84] Ramirez PT, Frumovitz M, Pareja R, Lopez A, Vieira M, Ribeiro R, et al Minimally invasive versus abdominal radical hysterectomy for cervical cancer. New England Journal of Medicine. 2018. 10.1056/NEJMoa1806395 30380365

[85] Allemani C, Matsuda T, Di Carlo V, Harewood R, Matz M, Niksic M, et al Global surveillance of trends in cancer survival 2000–14 (CONCORD-3): analysis of individual records for 37 513 025 patients diagnosed with one of 18 cancers from 322 population-based registries in 71 countries. Lancet. 2018;391(10125):1023–75. Epub 2018/02/06. 10.1016/S0140-6736(17)33326-3 29395269 PMC5879496

[86] Ntekim A, Campbell O, Rothenbacher D. Optimal management of cervical cancer in HIV-positive patients: a systematic review. Cancer Med. 2015;4(9):1381–93. Epub 2015/07/03. 10.1002/cam4.485 26136407 PMC4567023

[87] Jacobs JP, Jacobs ML, Mavroudis C, Maruszewski B, Tchervenkov CI, Lacour-Gayet FG, et al What is operative morbidity? Defining complications in a surgical registry database. The Annals of Thoracic Surgery. 2007;84(4):1416–21.

[88] Johnson N, Barlow D, Lethaby A, Tavender E, Curr L, Garry R. Methods of hysterectomy: systematic review and meta-analysis of randomised controlled trials. Bmj. 2005;330(7506):1478 10.1136/bmj.330.7506.1478 15976422 PMC558455

[89] Gupta S, Maheshwari A, Parab P, Mahantshetty U, Hawaldar R, Sastri S, et al Neoadjuvant Chemotherapy Followed by Radical Surgery Versus Concomitant Chemotherapy and Radiotherapy in Patients With Stage IB2, IIA, or IIB Squamous Cervical Cancer: A Randomized Controlled Trial. Journal of Clinical Oncology. 2018;36(16):1548–55. 10.1200/JCO.2017.75.9985 . 29432076

[90] Schellekens P. A changing China: Implications for developing countries. World Bank-Economic Premise. 2013;(118):1–9.

[91] Allemani C, Weir HK, Carreira H, Harewood R, Spika D, Wang X-S, et al Global surveillance of cancer survival 1995–2009: analysis of individual data for 25 676 887 patients from 279 population-based registries in 67 countries (CONCORD-2). The Lancet. 2015;385(9972):977–1010. 10.1016/S0140-6736(14)62038-9 PMC4588097 25467588

[92] Dare AJ, Onajin-Obembe B, Makasa EM. A snapshot of surgical outcomes and needs in Africa. The Lancet. 2018;391(10130):1553–4. 10.1016/S0140-6736(18)30002-3 29306588

[93] Watters DA, Guest GD, Tangi V, Shrime MG, Meara JG. Global surgery system strengthening: it is all about the right metrics. Anesthesia & Analgesia. 2018;126(4):1329–39. 29547428 10.1213/ANE.0000000000002771

[94] Ginsburg O, Badwe R, Boyle P, Derricks G, Dare A, Evans T, et al Changing global policy to deliver safe, equitable, and affordable care for women’s cancers. The Lancet. 2017;389(10071):871–80. 10.1016/S0140-6736(16)31393-9 27814964

[95] World Health Organization. Making every baby count: audit and review of stillbirths and neonatal deaths. 2016.

[96] Fuxiang L, Huashu Li, Zhaoxia Mo. Therapeutic effect of preoperative interventional therapy on IB2~IIB cervical cancer. Chinese Oncology Clinical,. 2008;35(20):1165–7.

[97] Yaomei M, Guiling Z, Wei Y, Peisong S, Caiyan L, Fengxia X. Preoperative adjuvant chemotherapy and radiotherapy combined with radical hysterectomy for the treatment of 50 cases of stage I B2-IIA cervical cancer. Chinese Journal of Clinical Oncology. 2013;40(8):471–4

[98] Yao C. Comparison of curative effect between laparoscopic radical hysterectomy with pelvic nerve and traditional extensive hysterectomy. Journal of Dalian Medical University. 2015;1:49–52

