# Supplementary material for: Morbidity after surgical management of cervical cancer in low and middle income countries: A systematic review and meta-analysis
Source: PLoS One. 2019 Jul 3;14(7):e0217775. doi: 10.1371/journal.pone.0217775 (PMC6608935; doi:10.1371/journal.pone.0217775)
Supplement: S1 Table — (DOCX) [file pone.0217775.s001.docx]

S1 Table. Complete search strategy

| **Database** | **Search strategy** | **Results** |
| --- | --- | --- |
| Pubmed | Uterine Cervical Neoplasms/surgery*[MeSH] AND (((Hysterectomy/adverse effects*[MeSH]) OR (Laparoscopy/adverse effects[MeSH]) OR (Postoperative Complications*[MeSH]) OR (Operative Time[MeSH]) OR (Perioperative Period*[MeSH]) OR (Blood Loss, Surgical[MeSH]) OR (Intraoperative Complications[MeSH]) OR (Ureter[MeSH]) OR (Cystotomy[MeSH]) OR (Intestines/surgery[Mesh]) OR (Urinary Bladder, Neurogenic[Mesh]) OR (Lymphocele[Mesh]) OR (Fistula[Mesh])) OR (Hystere* OR Laparo* OR Complication* OR Operati* OR Blood OR Morbidity OR Bowel injury OR Pelvic abscess OR Vascular injury OR Lymph*)) | 3264 |
| Cochrane | cervical cancer | 48 |
| Cochrane Central Register of Controlled Trials | cervical cancer AND surgery | 244 |
| Embase | exp uterine cervix cancer/su [Surgery] AND ((infectious complication/ or complications.mp. or infection complication/ or neurological complication/ or postoperative complication/ or complication/ or wound complication/ or preoperative complication/) OR (hysterectomy/ or abdominal hysterectomy/ or radical hysterectomy/ or hysterectomy.mp.) OR (laparoscopy/ or laparoscopy.mp.) OR (exp operation duration/) OR (blood loss.mp. or exp bleeding/) OR (cystotomy.mp. or bladder perforation/ or cystotomy/ or cystostomy/) OR (exp intestine injury/ or bowel injury.mp.) OR (Pelvic abscess.mp. or pelvis abscess/) OR (exp blood vessel injury/co, su [Complication, Surgery]) OR (exp lymphocele/co, su [Complication, Surgery]) OR (exp fistula/co, su [Complication, Surgery])) | 4622 |
| LILACS | "UTERINE CERVICAL DISEASES" [Words] and ”SURGERY" [Words] | 13 |
| CINAHL | (MH "Cervix Neoplasms+")  AND ((MH "Hysterectomy") OR (MH "Hysterectomy, Vaginal") OR (MH "Laparoscopy") OR (MH "Surgery, Laparoscopic") OR (MH "Postoperative Complications") OR (MH "Postoperative Hemorrhage") OR (MH "Treatment Complications, Delayed") OR (MH "Postoperative Pain") OR (MH "Surgery, Operative") OR (MH "Blood Loss, Surgical") OR  (MH "Ureter") OR (MH "Cystotomy") OR (MH "Intestinal Perforation") OR (MH "Intestinal Fistula") OR (MH "Bladder, Neurogenic") OR (MH "Lymphocele") OR (MH "Fistula")) | 562 |
|  | TOTAL | 8740+13 |
|  | TOTAL WITH DUPLICATES REMOVED | 6903+13 |
