# Supplementary material for: Morbidity after surgical management of cervical cancer in low and middle income countries: A systematic review and meta-analysis
Source: PLoS One. 2019 Jul 3;14(7):e0217775. doi: 10.1371/journal.pone.0217775 (PMC6608935; doi:10.1371/journal.pone.0217775)
Supplement: S2 Table — (DOCX) [file pone.0217775.s002.docx]

**S2 Table. Sensitivity analysis for surgical complications reported in the meta-analysis**

| **Complication** | **Including China** | | | | **Excluding China** | | | | **Difference in random pooled estimate** |
| --- | --- | --- | --- | --- | --- | --- | --- | --- | --- |
|  | Random pooled estimate | 95% CI | I^2^  (%) | No. events | Random pooled estimate | 95% CI | I^2^  (%) | No. events |  |
| Blood transfusion | 0.29 | 0.19 – 0.41 | 97.8 | 1018/3208 | 0.36 | 0.26 – 0.47 | 88.0 | 297/806 | 0.07 |
| Nerve injury | 0.01 | 0.0 – 0.03 | 77.8 | 12/995 | 0.03 | 0.01 – 0.04 | 0.0 | 10/352 | 0.02 |
| Bowel injury | 0.01 | 0.00 – 0.01 | 0.0 | 12/1957 | 0.01 | 0.00 – 0.02 | 0.0 | 4/506 | - |
| Bladder injury | 0.01 | 0.01 - 0.02 | 32.2 | 68/4743 | 0.01 | 0.01 - 0.02 | 0.0 | 29/1896 | - |
| Ureteric injury | 0.01 | 0.01 - 0.01 | 0.00 | 61/5239 | 0.01 | 0.01 - 0.02 | 0.0 | 19/1749 | - |
| Vascular injury | 0.02 | 0.01 – 0.03 | 60.2 | 75/4327 | 0.02 | 0.01 – 0.04 | 0.0 | 20/751 | - |
| Fistula injury | 0.02 | 0.01 – 0.03 | 77.3 | 94/4381 | 0.04 | 0.02 – 0.05 | 33.5 | 61/1400 | 0.02 |
| Conversion to open surgery | 0.01 | 0.00 – 0.02 | 21.3 | 11/1015 | *All studies were from China* | | | | |
| Thromboembolic events | 0.01 | 0.00 – 0.02 | 0.0 | 18/1453 | 0.01 | 0.00 – 0.02 | 0.0 | 5/478 | - |
| Infectious morbidity | 0.08 | 0.04 – 0.12 | 95.7 | 434/4819 | 0.09 | 0.03 – 0.16 | 93.9 | 202/1546 | 0.01 |
| Overall prevalence of complications | 0.04 | 0.03 – 0.06 | 85.7 | 303/9185 | 0.05 | 0.02 – 0.08 | 90.1 | 92/3001 | 0.01 |

Abbreviation: CI – confidence interval
