# Supplementary material for: Morbidity after surgical management of cervical cancer in low and middle income countries: A systematic review and meta-analysis
Source: PLoS One. 2019 Jul 3;14(7):e0217775. doi: 10.1371/journal.pone.0217775 (PMC6608935; doi:10.1371/journal.pone.0217775)
Supplement: S1 File — (DOCX) [file pone.0217775.s003.docx]

**S1 File. All reported complications in included studies**

Adverse bowel function, Bladder atony, Blood loss, Blood transfusions, Bowel injury, Bowel obstruction, Carotid artery thrombosis, Cervical stenosis, Constipation, Conversion to open surgery, Death, Diarrhoea, DVT, Dyspareunia, Dysuria, Faecal incontinence, Febrile morbidity, Fistula, Flatus incontinence, Frequency / urgency, Haematoma, Hydronephrosis, Hypercapnia, Ileus, IUD withdrawal difficulty, Length of catheterisation, Length of stay, Local relapse, Lymphocyst, Mean time to voiding, Metabolic complication, Necrosis of vaginal fornix ,Necrotising fasciitis ,Nerve injury, Nocturia, Oedema/lymphadenopathy, Pelvic abscess, Pelvic cellulitis, Pneumonia, Port site hernia, Post op infection, Post-operative bleeding, Post-irradiation ileitis, Pulmonary embolism, Pyelonephritis, Quality of sexual life function, Radiation cystitis, Return to theatre, Satisfaction of urination, Satisfaction of defecation, Stress incontinence / urinary incontinence, Thrombophlebitis, Time to first bowel movement, Time to post-operative flatus, Ureterostenosis, Urinary retention, Urinary tract injury, Urosepsis, UTI, Vaginal bleeding ,Vaginal cuff infection, Vaginal tear, Vascular injury, Vault dehiscence, Wound dehiscence, Wound infection.
