# Supplementary material for: Morbidity after surgical management of cervical cancer in low and middle income countries: A systematic review and meta-analysis
Source: PLoS One. 2019 Jul 3;14(7):e0217775. doi: 10.1371/journal.pone.0217775 (PMC6608935; doi:10.1371/journal.pone.0217775)
Supplement: S2 File — (DOCX) [file pone.0217775.s004.docx]

**S2 File. Funnel plots reporting surgical complications for all studies included in the meta-analysis**

| **Figure A** - Funnel plot (with pseudo 95% confidence limits), using data from 15 studies that reported on blood transfusions (p= 0.889). | **Figure B** - Funnel plot, using data from 4 studies reporting on nerve injury (p= 0.326). | **Figure C** - Funnel plot, using data from 6 studies reporting on bowel injury (p= 0.295). |
| --- | --- | --- |
| 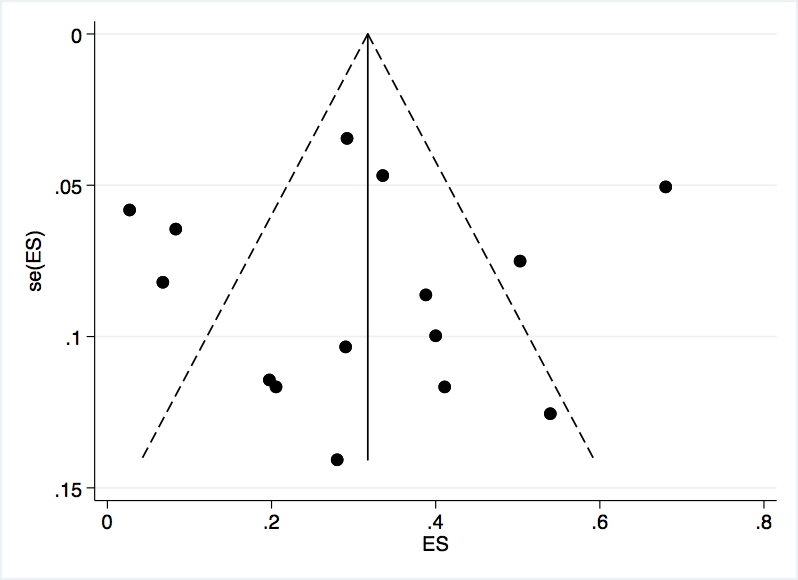 | 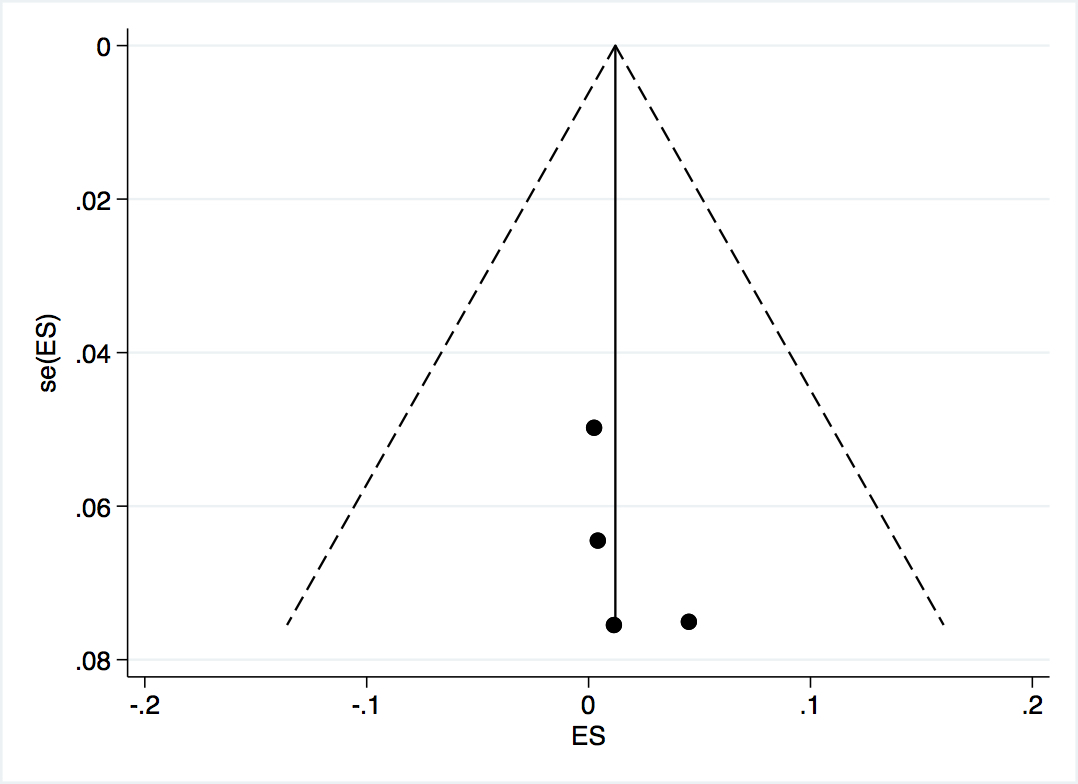 | 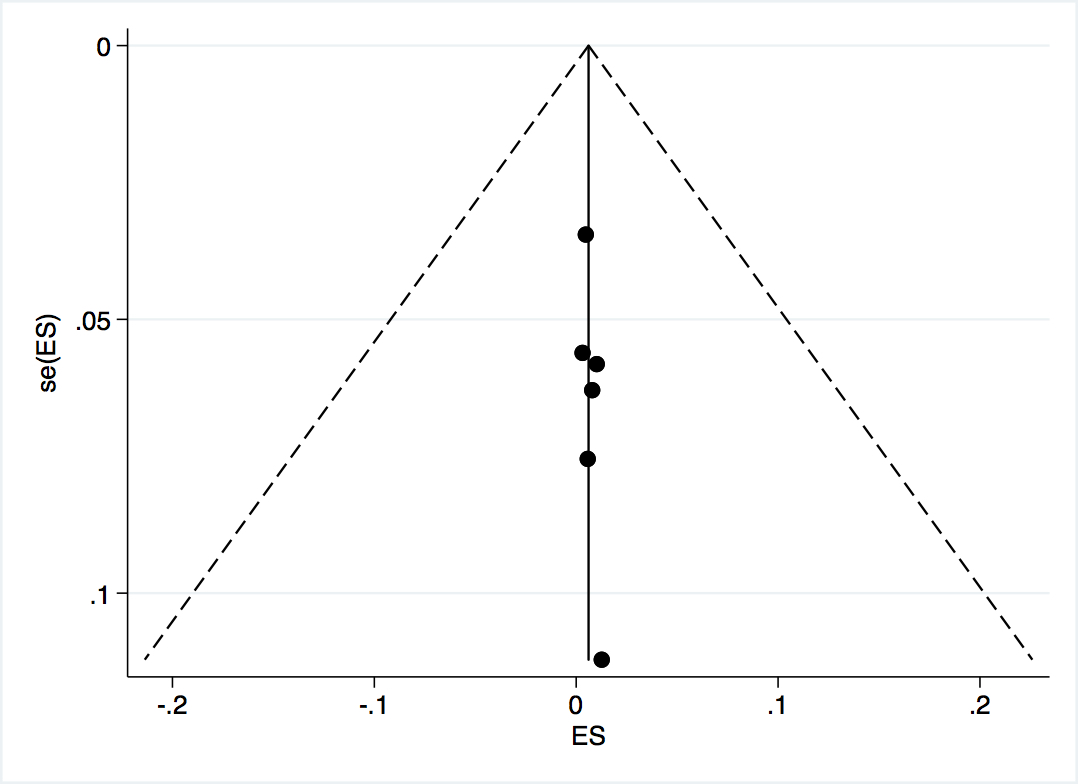 |
| **Figure D** - Funnel plot (with pseudo 95% confidence limits), using data from 16 studies reporting on bladder injury (p= 0.059). | **Figure E** - Funnel plot (with pseudo 95% confidence limits), using data from 14 studies reporting on ureteric injury (p= 0.321). | **Figure F** - Funnel plot, using data from 14 studies reporting on vascular injury (p= 0.091). |
| 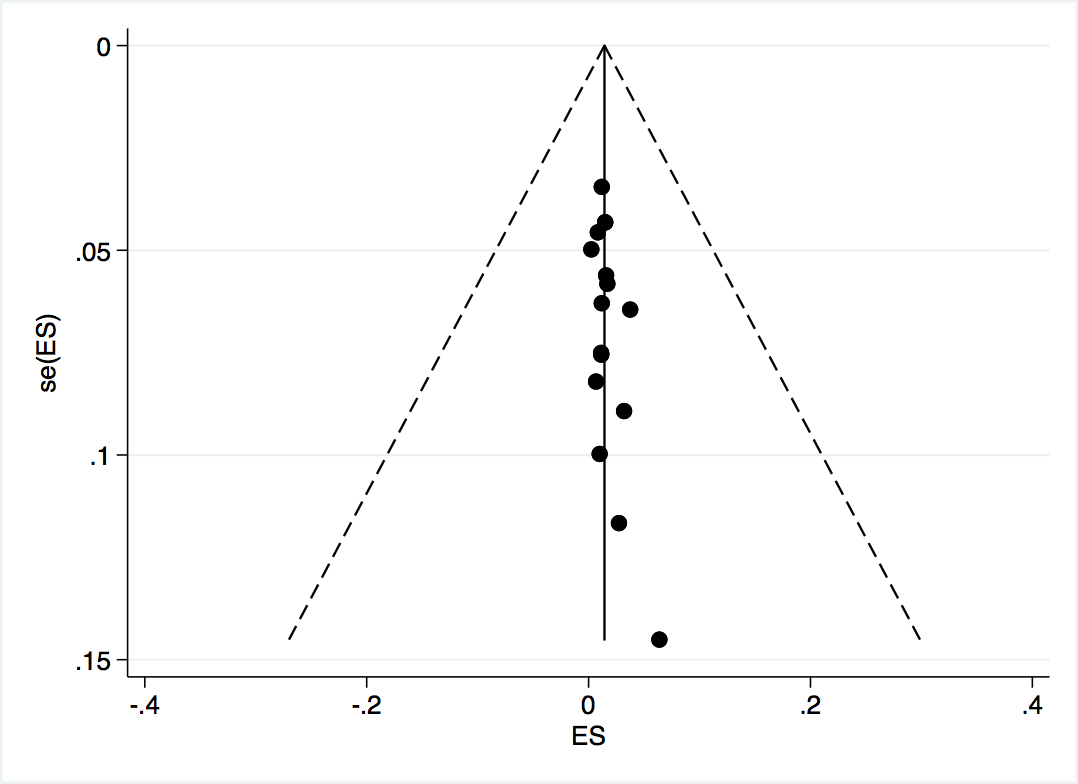 | 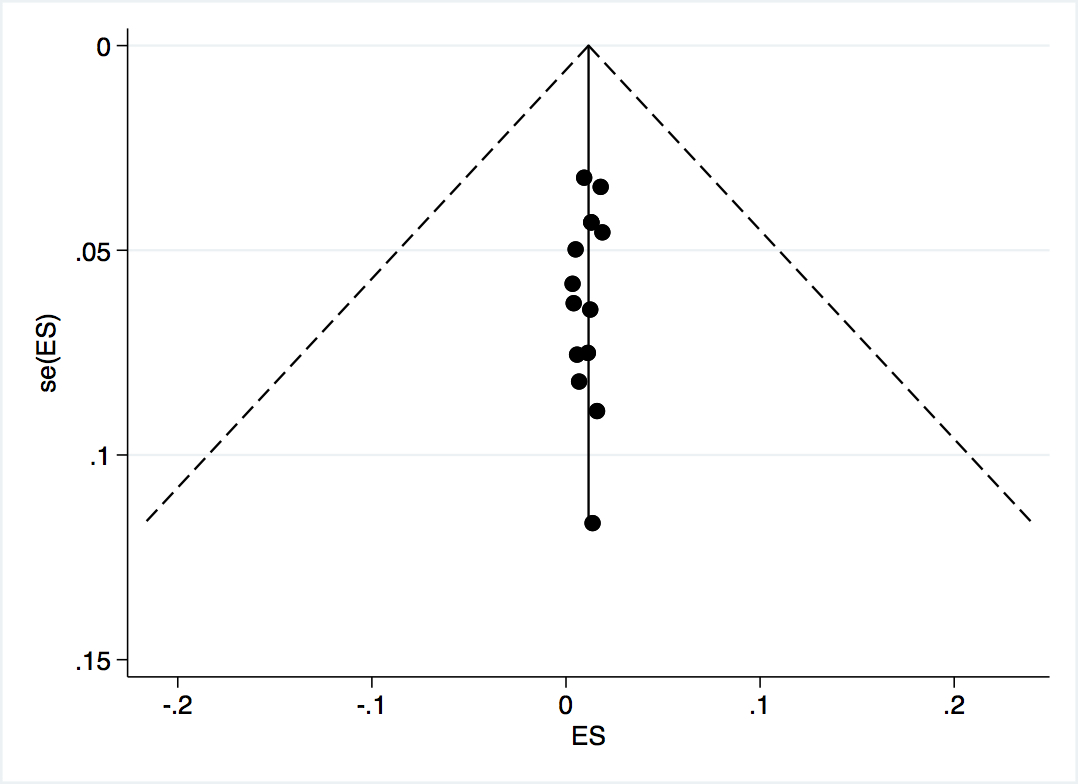 | 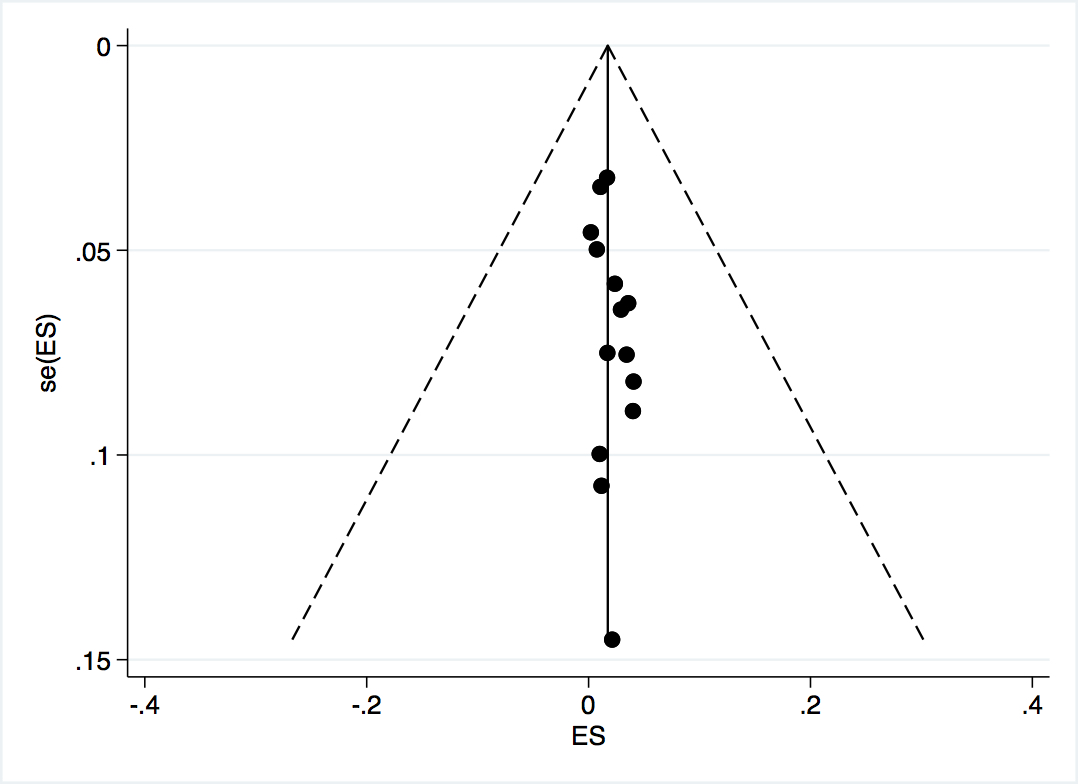 |

| **Figure G** - Funnel plot (with pseudo 95% confidence limits), using data from 14 studies reporting on fistula (p=0.811 ). | **Figure H** - Funnel plot (with pseudo 95% confidence limits), using data from 3 studies that reported on conversion to laparotomy (p= 0.077). | **Figure I** - Funnel plot, using data from 7 studies reporting on combined thromboembolic events (p=0.298). |
| --- | --- | --- |
| 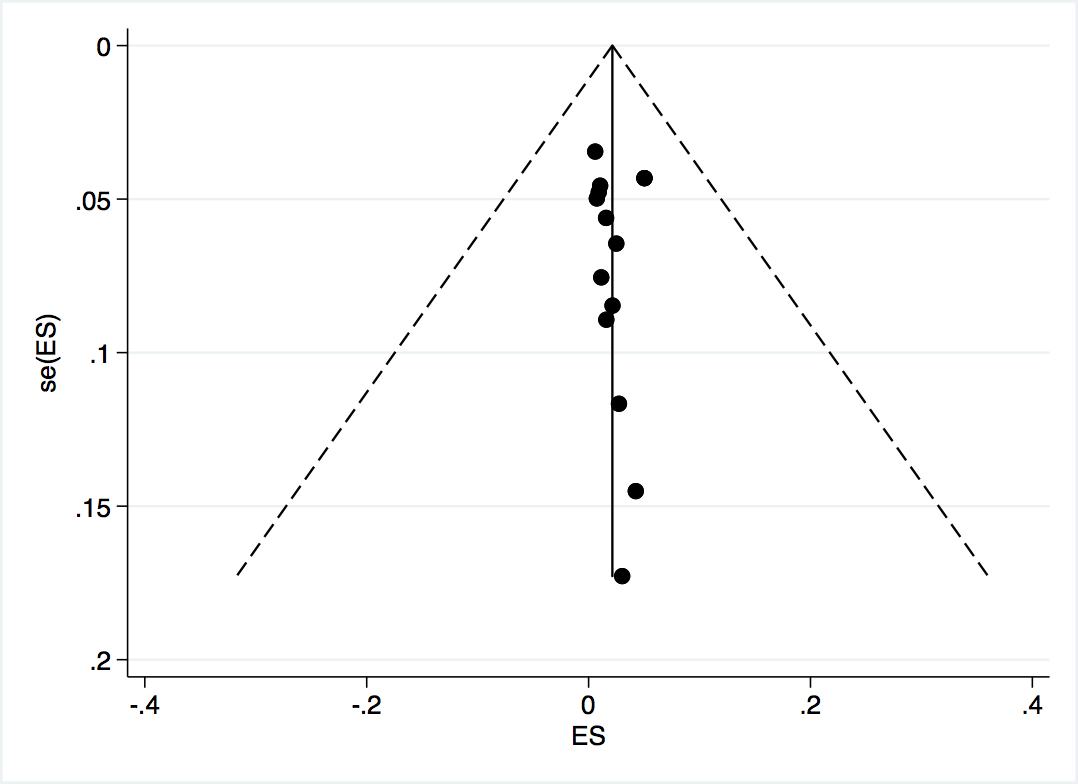 | 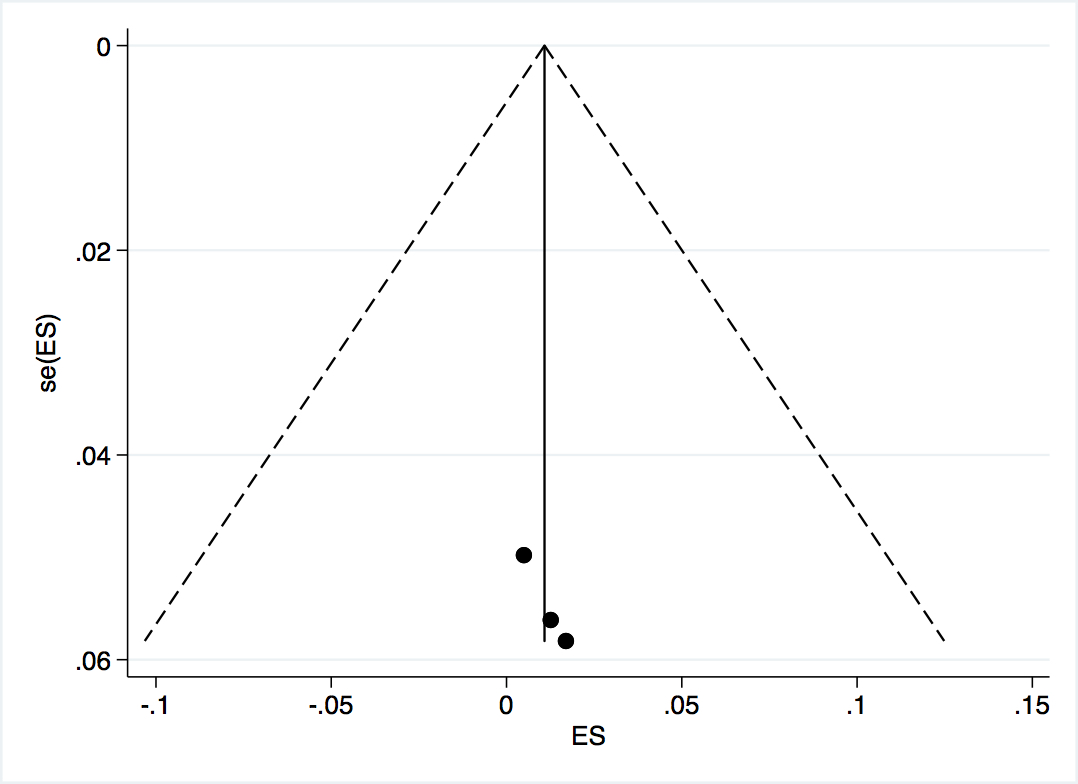 | 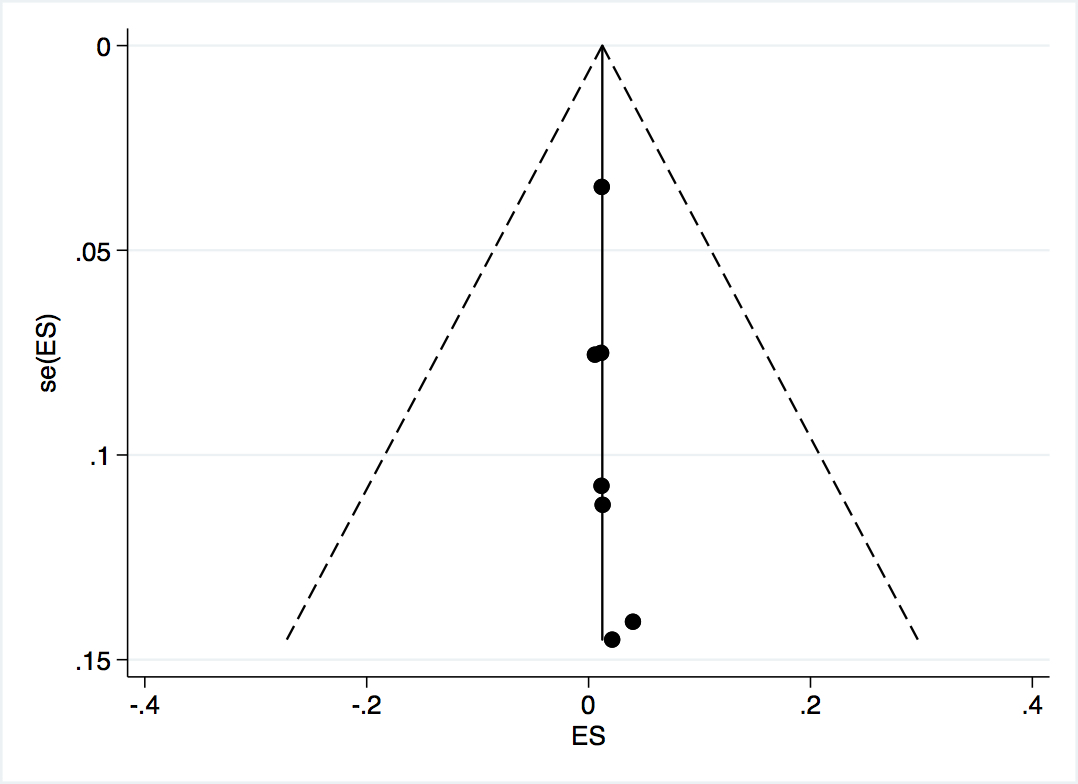 |
| **Figure J** - Funnel plot, using data from 20 studies reporting on infectious morbidity (p= 0.792). |  |  |
| 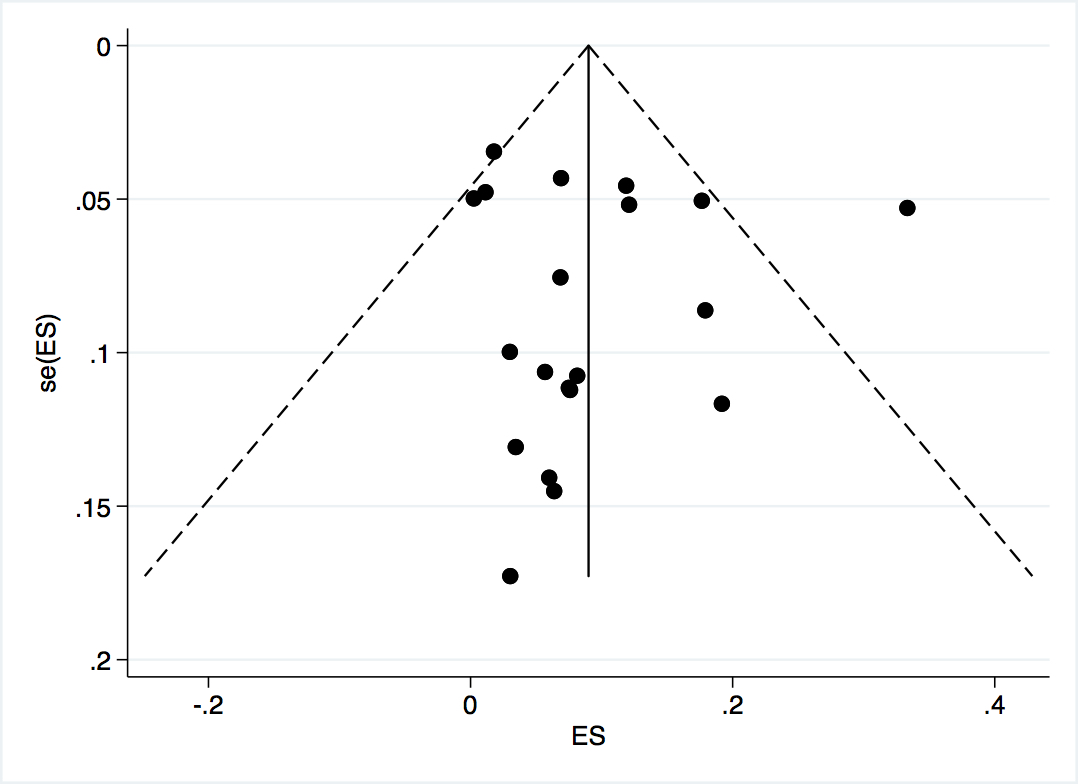 |  |  |
